# Macrophage reprogramming in degenerative musculoskeletal diseases: From tissue-niche biology to therapeutic translation

**DOI:** 10.1016/j.jot.2026.101244

**Published:** 2026-10-24

**Authors:** Chao Wang, Yuxin Chen, Dong Ren, Yufeng Chen, Guowei Ren, Tong Li, Mehboob Ullah, Qin Xia, Lei Dong, Pengcheng Wang

**Affiliations:** a Orthopaedic Trauma Service Center, Third Affiliated Hospital of Hebei Medical University, Shijiazhuang, 050011, China; b School of Life Science, Beijing Institute of Technology, Beijing, 100081, China

**Keywords:** Intervertebral disc degeneration, Macrophages, Musculoskeletal diseases, Osteoarthritis, Osteoporosis, Sarcopenia

## Abstract

Degenerative musculoskeletal diseases (DMSDs), including osteoarthritis (OA), osteoporosis (OP), intervertebral disc degeneration (IVDD), and sarcopenia (SP), impose a major burden through pain, structural deterioration, mobility loss, fracture risk, and functional decline. Although these disorders are often grouped as age-related musculoskeletal conditions, they arise from distinct tissue niches and cannot be explained by a single pathogenic mechanism. Macrophages occupy a central position at the interface between degenerative stress and tissue outcome, but their functions extend far beyond a binary M1/M2 polarization model. This Review proposes a disease-niche-adapted framework for macrophage reprogramming in DMSDs. In this framework, macrophages are interpreted according to the pathological tasks they perform within each tissue environment: inflammatory amplification and pain-related synovial crosstalk in OA, osteoimmune remodeling in OP, immune-barrier disruption and macrophage-nucleus pulposus (NP) cell feedback in IVDD, and impaired regenerative support in aging muscle in SP. We discuss how macrophage functional states are shaped by transcriptional and epigenetic regulation, immunometabolic rewiring, inflammatory signaling, autophagy and organelle quality control, and local niche-derived cues. Therapeutic strategies are then evaluated according to whether they interrupt upstream niche stress, redirect inflammatory and metabolic decoding, restore quality-control capacity, or reshape macrophage-mediated tissue communication. Finally, we propose that macrophage-targeted therapy should be assessed through a hierarchy of evidence, progressing from marker changes to functional reprogramming, macrophage-causal validation, and disease-specific structural or functional improvement. This framework may help convert macrophage plasticity from a broad immunological concept into a more precise, testable, and disease-adapted therapeutic strategy for musculoskeletal degeneration.

**The translational potential of this article:**

This Review provides a disease-niche-adapted framework for evaluating macrophage reprogramming in degenerative musculoskeletal diseases. By linking disease-specific macrophage tasks with regulatory mechanisms, therapeutic entry points, causal validation, delivery feasibility, and tissue-relevant endpoints, this framework may help guide both the design and translational assessment of macrophage-targeted interventions for musculoskeletal degeneration.

## Graphical abstract

**Figure:**
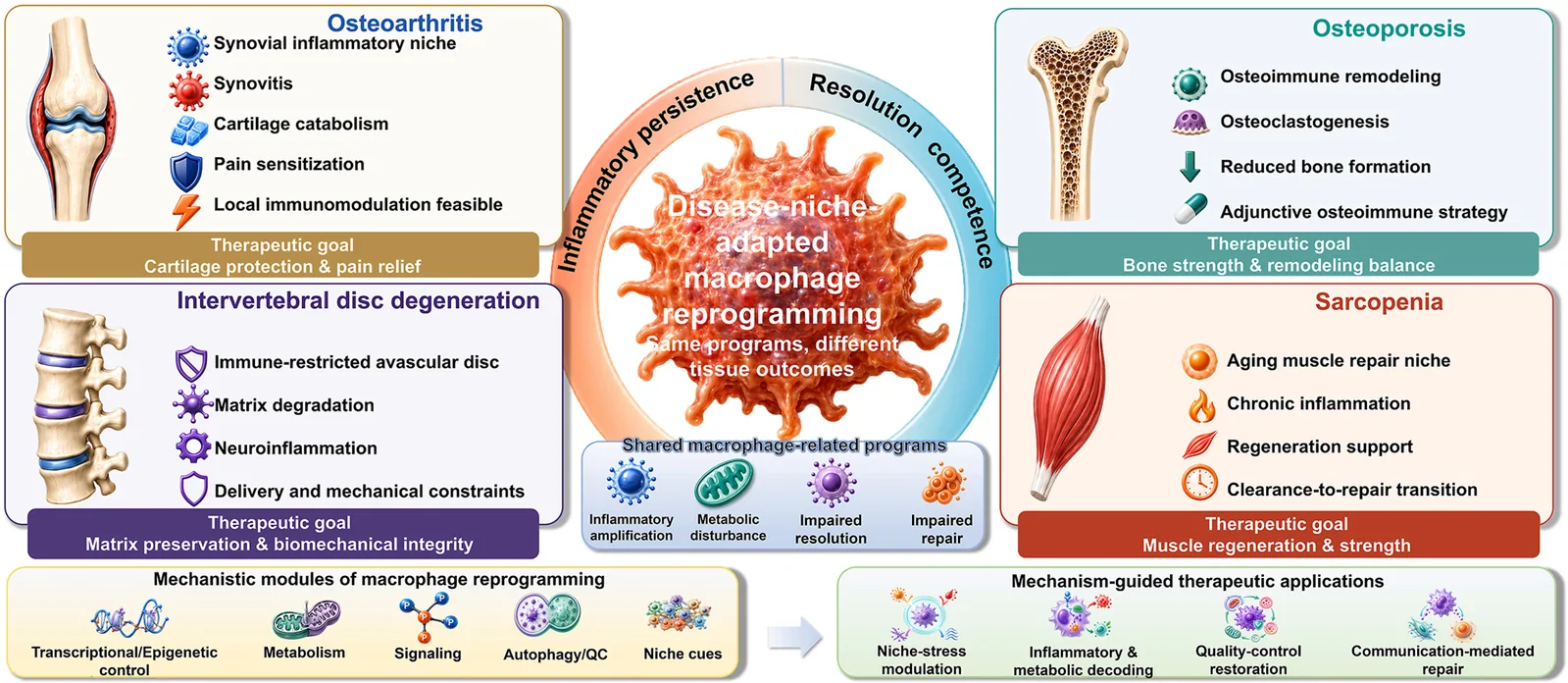

## 1 Introduction

Degenerative musculoskeletal diseases (DMSDs), including osteoarthritis (OA), osteoporosis (OP), intervertebral disc degeneration (IVDD), and sarcopenia (SP), represent a major component of the age-related disease burden. Together, these conditions lead to chronic pain, reduced mobility, increased fracture risk, loss of independence, and impaired quality of life. For this reason, they are often discussed as a group of interrelated musculoskeletal degenerative disorders [1], [2], [3], [4], [5], [6], [7], [8], [9]. However, placing these diseases within a shared category does not mean that they arise from the same tissue environment or follow a unified pathogenic mechanism. OA develops within the synovial joint environment and is characterized by cartilage degeneration, synovial inflammation, subchondral bone remodeling, and pain sensitization. OP reflects a systemic imbalance between bone resorption and bone formation. IVDD occurs within a specialized tissue that is relatively avascular, nutrient limited, and immune restricted. SP involves aging-associated muscle loss, physical inactivity, neuromuscular junction degeneration, mitochondrial dysfunction, and systemic metabolic disturbance [10], [11], [12], [13], [14], [15], [16], [17], [18], [19], [20], [21], [22], [23], [24], [25], [26], [27], [28], [29]. Thus, although DMSDs share clinical and pathological contexts, they cannot be reduced to a single mechanism-driven disease spectrum.

Despite marked differences in anatomical location, cellular composition, and clinical presentation, these diseases share several deeper pathological features. A key commonality is that affected tissues remain trapped in maladaptive stress responses for prolonged periods. During this process, chronic low-grade inflammation, extracellular matrix (ECM) degradation, abnormal mechanical microenvironments, oxidative stress, cellular senescence, and metabolic disturbance reinforce one another, preventing tissues from transitioning effectively from injury response to repair [9], [30], [31], [32], [33], [34], [35]. Inflammation in degenerative tissues is therefore not merely a passive consequence of disease progression. More importantly, persistent inflammation reflects a failure to terminate damage-associated signals and a failure to initiate or sustain reparative programs. Unlike controlled inflammation after acute injury, chronic inflammation in degenerative tissues is often accompanied by insufficient apoptotic cell clearance, imbalanced matrix remodeling, persistent oxidative and metabolic stress, and weakened tissue-supportive repair responses.

Macrophages are positioned at a critical intersection between degenerative stimuli and tissue pathological outcomes. As highly plastic cells of the innate immune system, macrophages participate not only in inflammatory amplification, but also in debris clearance, efferocytosis, ECM remodeling, angiogenesis, neuroimmune regulation, and tissue repair. Their functional states are continuously shaped by the local tissue microenvironment, including cytokines, chemokines, matrix fragments, damage-associated molecular patterns, hypoxia, acidic pH, lipid mediators, mitochondrial stress, metabolic substrates, and mechanical signals [36], [37], [38], [39], [40], [41], [42]. Therefore, in degenerative diseases, macrophages should not be understood simply as pro-inflammatory or anti-inflammatory cells. They are better viewed as dynamic integrators of tissue niche signals, whose functional states reflect the local pathological environment while also amplifying, sustaining, or reshaping that environment.

Historically, macrophage responses in DMSDs have often been interpreted using the M1/M2 polarization framework, in which M1-like states are broadly associated with inflammatory programs and M2-like states with inflammation resolution and tissue remodeling [43], [44]. This framework remains useful for describing macrophage responses under defined experimental polarization conditions, but its explanatory value is more limited in vivo, where macrophage phenotypes are often mixed, dynamic, and strongly shaped by tissue context [45], [46], [47]. Increasing evidence therefore favors interpreting macrophage states in relation to their functional programs, cellular origin, spatial localization, and disease stage, rather than assigning them to fixed polarization categories. This distinction is particularly relevant in degenerative musculoskeletal tissues, where similar macrophage programs may have different consequences depending on the local niche and the regenerative demands of the tissue.

From this perspective, the significance of macrophage plasticity in DMSDs should be understood in terms of whether macrophages can promote termination of tissue stress, resolution of inflammation, and restoration of a repair-permissive microenvironment. In OA, synovial macrophages can amplify synovitis, cartilage catabolism, and pain sensitization through cytokines, chemokines, and matrix-degrading enzymes [48], [49], [50], [51], [52], [53], [54], [55], [56]. In OP, macrophages are embedded within the osteoimmune remodeling network and contribute to the balance between bone resorption and bone formation [57]. Pro-inflammatory macrophage programs amplify osteoclastogenesis through TNF-α, IL-1, IL-6, RANKL-related signaling and M-CSF-dependent osteoclast precursor expansion, while also impairing osteoblast proliferation and osteogenic differentiation [58], [59], [60], [61], [62], [63], [64], [65]. In IVDD, macrophage function is shaped by the immune-restricted and avascular disc niche, as well as by dense ECM and sustained mechanical loading [24], [25], [26], [27]. During degeneration, annulus fibrosus disruption, neurovascular ingrowth, and inflammatory signals released by disc cells promote macrophage infiltration, inflammatory amplification, matrix destruction, and discogenic pain [66], [67], [68], [69], [70], [71], [72]. In SP, macrophages operate within the immune-satellite cell-muscle remodeling axis. They support necrotic fiber clearance and early regeneration after injury, whereas aging-associated immune dysregulation can prolong inflammatory signaling, impair regenerative timing, and contribute to muscle wasting [29], [72], [73], [74], [75], [76]. These findings indicate that macrophage function is strongly dependent on disease context and tissue niche, and cannot be interpreted apart from specific tissue architecture, disease stage, and repair demand.

This Review therefore proposes a disease-niche-adapted framework for interpreting macrophage reprogramming in DMSDs. Rather than introducing another macrophage taxonomy, the framework builds on established concepts of macrophage polarization and plasticity [77], [78]. Recent high-resolution work has further emphasized that macrophage states are shaped by ontogeny, local tissue niches, and disease-stage-specific signals [45], [79], [80], [81], [82]. Building on these advances, the present framework uses the disease niche as the organizing unit and links macrophage state and function with spatial and temporal context, causal relevance, and therapeutic feasibility. It is intended to bridge the gap between descriptive macrophage phenotyping, disease-specific functional interpretation, causal validation, and therapeutic translation. The relationship between these conceptual approaches is summarized in Table 1.

**Table 1:** Comparison of macrophage conceptual approaches relevant to degenerative musculoskeletal diseases.

Conceptual approach	Main emphasis	What it captures well	Additional considerations in DMSDs	Implications for research and therapeutic development
M1/M2 polarization	Macrophage responses under defined polarizing conditions	Provides a practical vocabulary for describing experimentally induced inflammatory and alternative activation programs	In vivo macrophages frequently display mixed, dynamic, and context-dependent states that are not fully represented by binary labels	Useful for descriptive phenotyping, but marker changes should be complemented by functional and disease-relevant outcome assessment
Macrophage plasticity and multidimensional concepts	Macrophage states shaped by ontogeny, activation history, and environmental signals	Captures macrophage heterogeneity, dynamic state transitions, and the influence of tissue context beyond binary polarization	Disease relevance depends on determining where a particular macrophage state occurs, what function it performs, at which disease stage, and whether it contributes causally to tissue outcome	Encourages multidimensional, spatial, and functional characterization and provides the biological basis for context-dependent interpretation of macrophage states
Disease-niche-adapted framework used in this Review	Integration of macrophage state and function with the specific disease niche and translational context	Organizes macrophage biology according to disease-specific pathological tasks, regulatory mechanisms, causal evidence, therapeutic feasibility, and tissue-level outcomes	Requires disease- and stage-specific evidence and does not assume that the same macrophage state or pathway has equivalent consequences across different tissues	Guides experimental design toward spatially and stage-resolved functional and causal validation, and therapeutic development toward disease-relevant macrophage functions, delivery feasibility, preservation of neighboring cells, and meaningful structural or functional outcomes

The framework is organized across three linked layers: disease-niche-specific macrophage functions, the regulatory mechanisms that generate these functions, and therapeutic strategies evaluated according to macrophage dependence, delivery feasibility, and disease-relevant tissue outcomes. In experimental design, this approach shifts emphasis from marker-defined macrophage states toward identifying the relevant macrophage population in its spatial and disease-stage context, testing its disease-relevant function, and establishing whether that function is causally linked to tissue degeneration or repair. In therapeutic development, it shifts emphasis from generic macrophage repolarization or pathway inhibition toward selecting disease-relevant macrophage functions and determining whether they can be modified within the anatomical, cellular, and delivery constraints of the affected niche while producing meaningful tissue-level benefit.

## 2 Macrophage niches and functional boundaries across distinct degenerative musculoskeletal diseases

Although OA, OP, IVDD, and SP share chronic inflammation and impaired tissue repair, the macrophage compartment is organized differently across these disease niches. In OA, resident synovial macrophages coexist with recruited monocyte-derived populations within a relatively accessible joint environment, where macrophages participate in synovitis, cartilage catabolism, subchondral remodeling, and pain-related signaling [82]. In OP, bone marrow macrophages and osteomacs operate within an osteoimmune remodeling system that overlaps with the monocyte-osteoclast lineage continuum; their disease relevance is therefore closely linked to the balance between bone resorption and formation, while therapeutic macrophage modulation is more appropriately positioned as an adjunct to bone-directed or regenerative treatment [57]. In IVDD, macrophage abundance and localization are strongly stage-dependent because immune-cell access increases as the normally avascular and immune-restricted disc becomes structurally disrupted; these macrophages can promote matrix degradation, nucleus pulposus (NP)-cell dysfunction, and discogenic pain, whereas the dense ECM, confined anatomy, and continuous mechanical loading substantially restrict therapeutic access [26], [27]. In SP, macrophages participate in a temporally coordinated transition from debris clearance to regenerative support within an aging muscle environment shaped by systemic inflammation, metabolic dysfunction, and impaired regenerative capacity; however, the developmental origin of chronic sarcopenia-associated macrophage states remains incompletely resolved, and therapeutic modulation must preserve the physiological macrophage functions required for muscle repair [75], [83]. Thus, differences in macrophage origin, tissue microenvironment, pathological function, and therapeutic accessibility provide the comparative basis for interpreting macrophage reprogramming across DMSDs and for the disease-specific sections that follow.

This niche-based interpretation has been sharpened by the increasing resolution of single-cell and spatial omics, which reveal macrophage heterogeneity that is obscured in bulk tissue analyses [84]. These approaches provide complementary information: single-cell or single-nucleus transcriptomics resolves macrophage states and potential interaction networks, whereas spatial approaches establish their anatomical context. Developmental origin requires additional lineage-tracing or fate-mapping evidence. Throughout this Review, macrophage state, spatial localization, and cellular origin are therefore considered related but distinct dimensions of heterogeneity.

### 2.1 Osteoarthritis: the synovial-chondral-subchondral axis

OA is a whole joint disease characterized by cartilage matrix degradation, synovial inflammation, subchondral bone remodeling, osteophyte formation, and pathological changes in periarticular tissues, including the meniscus, ligaments, and infrapatellar fat pad [10], [11], [12]. The pathological relevance of macrophages in OA is closely shaped by the synovial joint niche. Within this environment, synovial macrophages are continuously exposed to cartilage debris, damage-associated molecular patterns, inflammatory mediators, and mechanical stress. They also engage in dynamic crosstalk with chondrocytes, synovial fibroblast-like cells, osteoclasts, endothelial cells, and sensory nerve fibers. Therefore, macrophages in OA should not be viewed as a single initiating driver of degeneration, but rather as critical amplifiers linking joint inflammation, cartilage catabolism, subchondral bone remodeling, and pain sensitization. In healthy synovium, resident macrophages contribute to immune surveillance, apoptotic cell clearance, and maintenance of the synovial barrier [48], [49]. During the onset and progression of OA, cartilage matrix fragments, GM-CSF, IFN-γ, IL-1β, TNF-α, and related signals disrupt this homeostatic state, promote peripheral monocyte recruitment, and drive synovial macrophages toward pro-inflammatory functional programs [50]. Through NF-κB, MAPK, and other signaling pathways, these macrophages secrete TNF-α, IL-1β, IL-6, chemokines, and matrix-regulatory factors, thereby enhancing chondrocyte catabolic activity and inducing the expression of matrix metalloproteinases (MMPs) and a disintegrin and metalloproteinase with thrombospondin motifs (ADAMTS) family members. This process establishes a self-reinforcing loop between synovitis and cartilage matrix destruction [51]. Macrophage-mediated pathology in OA extends beyond cartilage damage. Macrophage-derived R-spondin 2 can activate Wnt/β-catenin signaling in chondrocytes and enhance matrix-degrading enzyme expression [85]. Macrophages can also accumulate in the infrapatellar fat pad, synovial fluid, and subchondral bone, where they promote osteoclast differentiation, aberrant bone remodeling, and osteophyte formation [52], [53], [54], [55], [56], [86].

At the same time, macrophage programs associated with inflammation resolution are equally important for restraining synovitis and preserving joint homeostasis. Macrophage states marked by IL-10, TGF-β, Arg1, CD206, and related molecules can suppress inflammatory cytokine production, limit cartilage catabolism, and support tissue stabilization [48], [49]. However, under persistent mechanical stress and inflammatory stimulation, these protective programs are often insufficient to counterbalance the pro-inflammatory amplification circuit. Therefore, macrophage modulation in OA therapy should be understood as a local immune regulatory strategy within the synovial joint cavity, aiming to promote inflammation resolution, reduce pain-related inflammation, and preserve essential clearance functions and matrix homeostasis.

In OA, this heterogeneity appears to reflect a reorganization of the synovial macrophage ecosystem rather than a simple shift between inflammatory and anti-inflammatory states. Monocyte-independent TIM4+CX3CR1+ macrophages and recruited monocyte-derived macrophages occupy different biological and anatomical positions, and experimental OA is accompanied by expansion of the recruited compartment together with redistribution of resident macrophages within the synovium [87]. These populations also participate in distinct stromal interactions. In human OA, a CCR2-enriched recruited macrophage population has been linked to fibroblast activation through oncostatin M (OSM)-OSM receptor (OSMR) signaling, while single-cell and spatial analyses have identified inflammatory, pain-associated, and fibrotic macrophage programs within distinct joint and synovial niches [88], [89]. Thus, the significance of macrophage heterogeneity in OA lies not simply in the presence of additional transcriptional clusters, but in the coexistence and spatial reorganization of macrophage populations with different origins and tissue interactions. This distinction may help explain why broadly suppressing or “repolarizing” synovial macrophages is unlikely to reproduce the effects of selectively modifying disease-associated macrophage-stromal circuits.

Taken together, these functional and cellular data make OA one of the most suitable DMSDs for macrophage-directed immunomodulation, because the joint cavity is relatively accessible, synovial macrophages are closely linked to disease phenotypes, and local delivery is clinically feasible. Nevertheless, even in OA, macrophage modulation cannot replace comprehensive management of mechanical loading, chondrocyte senescence, subchondral bone remodeling, and inflammatory phenotypes of individual patients. Its true value lies in providing a practical immune microenvironmental entry point for therapy that modifies disease progression.

### 2.2 Osteoporosis: macrophages in the osteoimmune remodeling axis

OP is an age-related skeletal disorder characterized by deterioration of bone microarchitecture, reduced bone mineral density, and increased fracture risk [13], [14], [15], [16]. Although its etiologies include postmenopausal, age-related, idiopathic, and secondary forms, the common pathological outcome is an imbalance in which bone resorption exceeds bone formation [17], [18], [19], [20], [21], [22], [23], [90], [91]. Macrophage function in OP should therefore be interpreted within the broader osteoimmune remodeling network rather than solely by the intensity of inflammation.

It is important to distinguish macrophage reprogramming from direct osteoclast inhibition in OP. Although osteoclasts arise from the monocyte-macrophage lineage, bone marrow macrophages, osteomacs, osteoclast precursors, and mature osteoclasts represent different functional states within the osteoimmune remodeling system [57]. Studies showing reduced osteoclast formation or decreased osteoclast-related markers should not automatically be interpreted as macrophage reprogramming unless macrophage-specific functions are also demonstrated. In the osteoporotic microenvironment, pro-inflammatory macrophage programs can promote bone resorption and suppress bone formation through mediators such as TNF-α, IL-1, and IL-6 [58], [59], [60], [61], [62], [63], [64]. Conversely, macrophage programs associated with inflammation resolution and tissue repair may restrict excessive osteoclast differentiation and support osteogenic differentiation through factors such as IL-4, IL-13, TGF-β, and BMP-2 [92], [93], [94].

A related but distinct issue arises in OP, where macrophage heterogeneity must be interpreted within the broader myeloid-osteoclast continuum. Single-cell analyses indicate that the osteoporotic myeloid compartment comprises multiple transcriptional states associated with different aspects of bone remodeling. Some macrophage populations are more closely associated with inflammatory regulation, whereas others show stronger relationships with osteoclast-related programs and intercellular signaling. For example, an OLR1-expressing macrophage population identified in osteoporosis was enriched for inflammatory and osteoclast-associated programs, with predicted heparin-binding EGF-like growth factor (HBEGF)-CD9 communication with osteoclasts [95]. The main implication is therefore not the identification of another marker-defined macrophage subset, but the need to resolve which myeloid populations regulate the bone-remodeling niche without conflating macrophage reprogramming with osteoclast differentiation. Because these assignments are based largely on transcriptional states and inferred trajectories, their developmental relationships still require direct lineage validation.

Hormonal changes, aging, mechanical unloading, metabolic disturbance, oxidative stress, and osteoblast dysfunction all contribute substantially to OP. Thus, macrophage reprogramming in OP should be positioned as a strategy to reshape the osteoimmune niche, rather than as a substitute for antiresorptive or osteoanabolic therapy.

Current OP treatment is dominated by antiresorptive agents such as bisphosphonates and denosumab, while selected osteoanabolic agents can stimulate bone formation [96], [97], [98], [99], [100]. Macrophage-related interventions may therefore be more appropriately developed as adjunctive osteoimmune strategies combined with antiresorptive therapy, osteoanabolic therapy, implant integration, or bone regenerative materials [101], [102], [103]. In postmenopausal or age-related OP, macrophage modulation may help alleviate inflammaging and osteoimmune imbalance. In secondary OP caused by endocrine, inflammatory, renal, hematological, or medication-related factors, macrophage modulation must be integrated with control of the primary disease mechanism [17], [18], [19], [20], [21], [22], [23], [90], [91]. In this sense, OP illustrates both the therapeutic potential and the conceptual boundary of macrophage-based immunomodulation: it is most meaningful when linked to bone remodeling balance and skeletal structural improvement, rather than to macrophage marker changes alone.

### 2.3 Intervertebral disc degeneration: macrophages in the axis of immune barrier disruption, matrix degradation, and neuroinflammation

IVDD is a degenerative spinal disorder characterized by ECM degradation, loss of disc height, annulus fibrosus rupture, nucleus pulposus (NP) cell dysfunction, endplate remodeling, and discogenic pain [24], [25]. Unlike joints, bone, and muscle, the healthy intervertebral disc is a relatively avascular and immune-restricted structure, composed primarily of the nucleus pulposus, annulus fibrosus, and cartilaginous endplate [26]. This specialized niche determines the strongly stage-dependent role of macrophages in IVDD. Immune cells are more likely to enter the inner disc only when annulus fibrosus rupture, neurovascular ingrowth, or disruption of the local inflammatory barrier occurs [27]. Degenerative NP and annulus fibrosus cells release inflammatory cytokines and chemokines that promote macrophage recruitment and activation [66]. Immunohistochemical studies have detected macrophage-associated markers such as CD197, CD163, and CD206 in degenerative intervertebral discs. The expression of some of these markers increases with the severity of degeneration, whereas healthy discs usually lack these features of immune cell infiltration [67], [104]. These findings support the involvement of macrophages in IVDD, while also indicating that their abundance, spatial distribution, and functional states may vary according to the patient, degenerative stage, and anatomical region of the disc.

IVDD presents a different pattern of macrophage heterogeneity because immune-cell access itself changes as degeneration progresses. Rather than indicating a stable macrophage population that is uniformly present throughout disease, available data suggest that macrophage abundance and state vary as the disc becomes progressively more permissive to immune-cell entry. Human single-cell studies show expansion of monocyte/macrophage populations with degeneration and identify disease-associated states such as SPP1-enriched macrophages, while more recent work has identified GPNMB^+^ macrophages that can directly influence NP-cell osteogenic reprogramming through platelet-derived growth factor (PDGF) signaling [105], [106]. Spatial transcriptomic analysis in experimental IVDD further demonstrates marked spatial and temporal remodeling of cellular stress and immune responses during degeneration [107]. Together, these findings suggest that macrophage heterogeneity in IVDD is shaped by both structural access to the disc and subsequent adaptation to distinct local microenvironments. A macrophage located near the outer annulus, endplate, or neurovascularized region may therefore not be biologically equivalent to one interacting directly with NP cells in advanced degeneration. The developmental origin and precise spatial organization of these populations in human IVDD, however, remain incompletely resolved.

Despite this heterogeneity, inflammatory macrophage programs can converge on several common mechanisms that accelerate disc degeneration. They produce MMPs, ADAMTS, and inflammatory cytokines, which promote ECM breakdown and weaken the mechanical stability of the disc. Macrophage-derived oncostatin M can activate the STAT3/YAP1 axis and influence osteoblast differentiation in the endplate region, thereby contributing to endplate remodeling [68]. In addition, macrophage-released prostaglandin E2, nerve growth factor, nitric oxide, and inflammatory chemokines can act on neural structures such as the dorsal root ganglion and sciatic nerve, promoting discogenic pain [69], [70]. A reciprocal inflammatory senescence loop also exists between macrophages and NP cells. Macrophage-mediated inflammatory signaling can induce NP cell senescence, disrupt cellular homeostasis, and reduce matrix synthetic capacity. In turn, senescent NP cells release senescence-associated secretory phenotype (SASP)-like factors that further attract and activate macrophages, forming a self-sustaining inflammatory degenerative circuit [71], [72].

However, IVDD is not driven solely by immunity. Mechanical loading, nutrient deprivation, matrix aging, endplate calcification, cellular senescence, and genetic susceptibility collectively shape disease progression. Therefore, macrophage modulation in IVDD should be positioned as one component of a multitarget disc protective strategy. Macrophage programs that promote resolution of inflammation may help stabilize the degenerative disc microenvironment. For example, small extracellular vesicles derived from macrophages polarized under M2-inducing experimental conditions can protect NP cells from pyroptosis through the PTEN/NLRP3 pathway and reduce matrix-degradation signals [108]. However, the dense ECM, avascular environment, confined anatomical space, and continuous mechanical loading of the intervertebral disc impose substantial barriers to therapeutic translation. Systemic administration is unlikely to reach the disc efficiently, while local injection may compromise annulus fibrosus integrity and mechanical stability. The value of macrophage modulation in IVDD therefore depends on controlling inflammatory injury while preserving disc structure and biomechanical function, rather than on reducing inflammatory markers alone.

### 2.4 Sarcopenia: macrophages in aging muscle regeneration

SP is an age-related syndrome characterized by progressive loss of skeletal muscle mass, strength, and physical function, often leading to frailty, falls, and increased fracture risk [8], [109]. Compared with OA, OP, and IVDD, SP is not simply a localized structural degenerative disease. Instead, it is shaped by systemic aging, neuromuscular junction degeneration, endocrine alterations, mitochondrial dysfunction, malnutrition, reduced physical activity, and metabolic disturbance [28], [29]. Therefore, macrophage reprogramming in SP should be regarded as an emerging research direction rather than a fully established therapeutic axis.

Macrophages play temporally coordinated roles in skeletal muscle injury and repair. During the early phase of injury, inflammatory macrophages clear necrotic myofibers and cellular debris and initiate the regenerative response. Subsequently, macrophage programs associated with inflammation resolution support satellite cell differentiation, angiogenesis, and ECM remodeling [36], [73], [74], [75], [110], [111]. This transition from clearance to repair forms the biological foundation of healthy muscle regeneration. In aged muscle, however, this process may become delayed or imbalanced. Studies have shown that repair-associated macrophage subsets are reduced in aged muscle, whereas inflammatory macrophages persist for longer periods, contributing to chronic low-grade inflammation, impaired satellite cell function, and muscle atrophy [75], [112], [113], [114], [115].

In aging muscle, macrophage heterogeneity appears to reflect broader remodeling of the regenerative immune niche rather than persistence of a single inflammatory state. Human aging and sarcopenia are accompanied by changes in the relative abundance and transcriptional programs of several macrophage populations, including LYVE1-associated and lipid-associated states, together with altered macrophage-centered intercellular communication [83], [116]. These observations extend the conventional clearance-to-repair model by suggesting that aging changes not only the timing of macrophage responses but also the composition and tissue interactions of the macrophage compartment itself. The therapeutic implication is therefore not to eliminate one age-enriched macrophage subset, but to determine which alterations impair macrophage support for muscle satellite cell (MuSC) function, vascular homeostasis, and muscle regeneration. At present, however, the developmental origin and causal contribution of individual macrophage states to chronic sarcopenia remain incompletely defined.

The key issue in SP is whether aged muscle can complete the transition from inflammatory clearance to repair-supporting macrophage function. Although transient inflammation is required for early regeneration, persistent TNF-α/IL-6 signaling can impair satellite-cell differentiation and promote muscle catabolism [112], [113], [114], [115]. Thus, this temporal pattern should be viewed as a biological constraint of regeneration rather than evidence for an established timing-based macrophage therapy.

Macrophage programs associated with inflammation resolution can support satellite cell differentiation, angiogenesis, and matrix remodeling through factors such as IL-10 and TGF-β [117], [118], [119]. However, in the aging microenvironment, these repair-supporting functions are often weakened by chronic inflammation, mitochondrial dysfunction, and metabolic stress. SP therefore provides an important reminder that macrophage modulation cannot be reduced to sustained anti-inflammatory polarization. Its therapeutic relevance should be tested by whether macrophages regain functional support for muscle regeneration. Compared with OA, OP, and IVDD, macrophage-targeted therapy in SP remains less mature and should be presented as an emerging regenerative immunology direction that still requires validation in chronic sarcopenia models using endpoints such as muscle mass, fibrosis, mitochondrial quality, neuromuscular integrity, grip strength, endurance, and functional recovery.

### 2.5 Cross-disease integration: resident-cell communication links macrophage state to tissue outcome

The disease-specific analyses above indicate that macrophage state becomes functionally relevant through the resident-cell network in which it operates. Macrophage-derived signals can alter stromal activation, matrix homeostasis, cell differentiation, nociceptive signaling, or regenerative activity, while resident cells can in turn recruit, activate, or reprogram macrophages. Cell-cell communication therefore provides a functional link between macrophage heterogeneity and tissue-level outcome.

The organization of these communication networks differs across DMSDs. In OA, macrophage interactions with synovial fibroblasts, chondrocytes, and sensory neural elements connect synovial inflammation with stromal activation, cartilage catabolism, and pain-related signaling [51], [85], [87], [88], [89]. In OP, macrophages communicate with osteoclast-lineage cells, osteoblasts, and mesenchymal stromal cells within the bone-remodeling unit, with both inferred macrophage-osteoclast ligand-receptor interactions and functionally supported macrophage-derived signals linking inflammatory macrophage states to increased bone resorption and impaired osteogenesis [58], [59], [60], [61], [62], [63], [64], [92], [93], [94], [95], [120], [121]. In IVDD, macrophage communication involves NP, AF, and endplate cells and becomes increasingly relevant as structural disruption permits immune-cell access to the normally immune-restricted disc [66], [68], [71], [72], [105], [106], [107], [108], [122]. These interactions are frequently reciprocal, because degenerative disc cells can promote macrophage recruitment and activation, whereas infiltrating macrophages subsequently modify disc-cell state and matrix homeostasis. In aging muscle, macrophages interact with satellite and other myogenic cells, fibro-adipogenic progenitors, and vascular cells within a regenerative network that coordinates debris clearance, myogenesis, stromal remodeling, and vascular support [73], [74], [75], [110], [111], [123].

Several features distinguish these networks beyond the identity of the interacting cells. Communication may be unidirectional or reciprocal, its dominant cellular targets can change with disease stage, and the same macrophage program can generate different consequences depending on the receiving cell and anatomical context. The strength of evidence also varies: some interactions are supported by direct functional experiments, whereas others are inferred primarily from single-cell, spatial, or ligand-receptor analyses. Fig. 1 therefore distinguishes representative functionally supported interactions from those that remain primarily inferential. Viewed at this network level, macrophage phenotype alone is insufficient to predict tissue effect; its biological significance depends on the macrophage population involved, the interacting resident cell, the direction of communication, spatial context, and the resulting functional outcome.

**Fig. 1:**
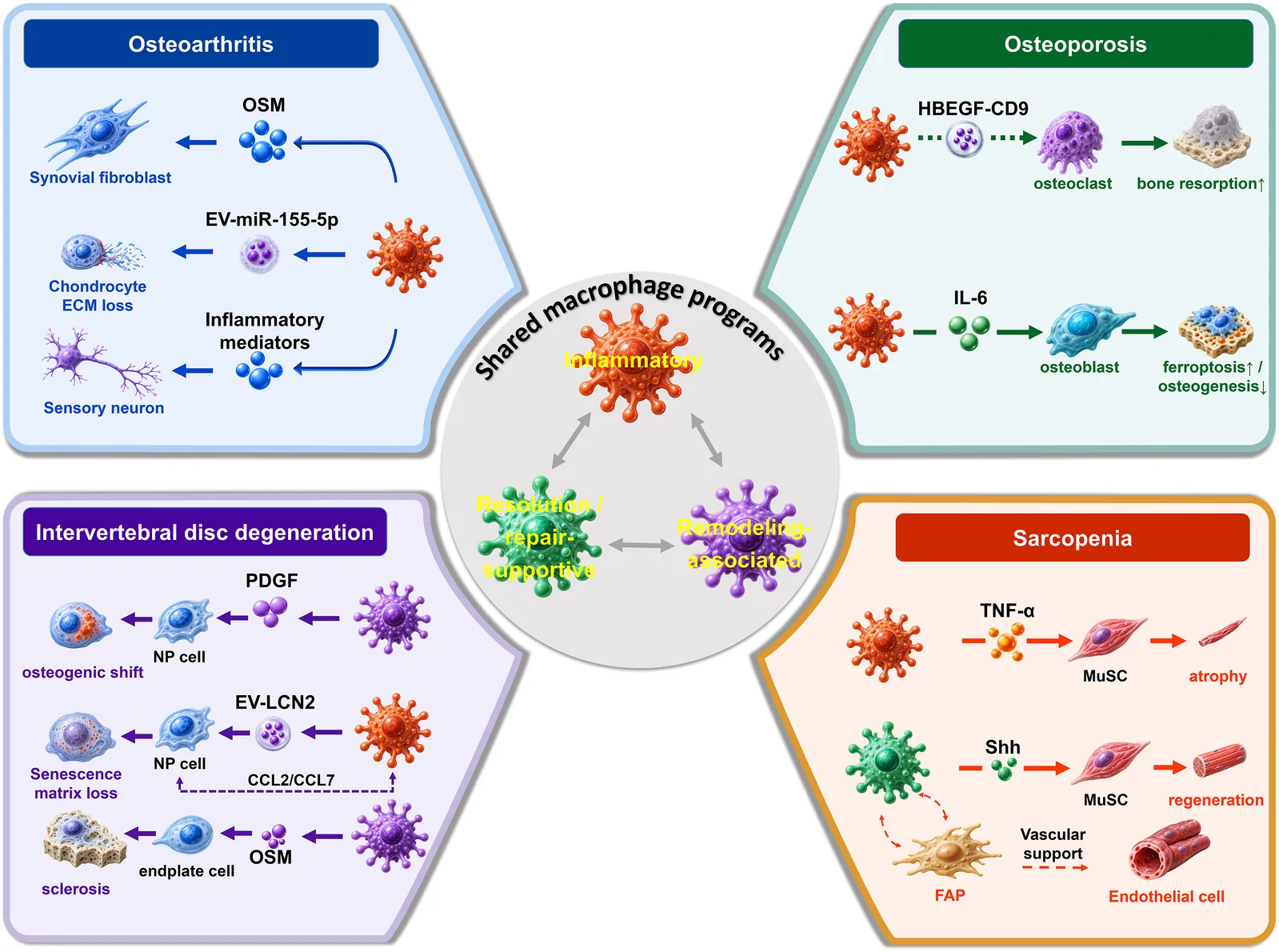
Macrophage-centered cell-cell communication across degenerative musculoskeletal diseases. Macrophage functional programs generate disease-specific tissue effects through communication with resident cells. In osteoarthritis (OA), macrophage-derived OSM, extracellular vesicles (EVs), and inflammatory mediators regulate synovial fibroblast activation, chondrocyte matrix catabolism, and sensory signaling. In osteoporosis (OP), macrophage-osteoclast communication involves predicted HBEGF-CD9 communication, whereas IL-6-mediated macrophage-osteoblast communication can impair osteogenesis and promote osteoblast dysfunction. In intervertebral disc degeneration (IVDD), macrophages communicate with nucleus pulposus (NP) and endplate cells through PDGF, EV-associated signals, OSM, and reciprocal chemokine-mediated recruitment, contributing to NP-cell reprogramming, senescence, matrix loss, and endplate remodeling. In sarcopenia (SP), inflammatory and repair-supportive macrophage signals influence myogenic cells, fibro-adipogenic progenitors (FAPs), and vascular cells, thereby modulating muscle atrophy, regeneration, and vascular support. The central schematic represents shared inflammatory, resolution/repair-supportive, and remodeling-associated macrophage programs rather than fixed polarization categories. Dashed arrows indicate reciprocal, predicted, or less directly validated interactions. Abbreviations: CCL2, C-C motif chemokine ligand 2; CCL7, C-C motif chemokine ligand 7; EV, extracellular vesicle; FAP, fibro-adipogenic progenitor; HBEGF, heparin-binding EGF-like growth factor; LCN2, lipocalin 2; MuSC, muscle satellite cell; OSM, oncostatin M; PDGF, platelet-derived growth factor; Shh, Sonic Hedgehog; CD9, cluster of differentiation 9; ECM, extracellular matrix; IL-6, interleukin-6; TNF-α, tumor necrosis factor-α.

## 3 Biological basis of macrophage functional reprogramming

Macrophage functional states in DMSDs emerge from the interaction between disease-niche signals and macrophage-intrinsic regulatory programs. Local cytokines, metabolites, hypoxia, oxidative stress, matrix-derived signals, and mechanical cues provide upstream inputs, whereas transcriptional and epigenetic regulation, immunometabolic rewiring, inflammatory signaling, and autophagy-organelle quality control determine how these inputs are sensed, integrated, and translated into functional responses. These intracellular regulatory layers are discussed separately below for clarity, but they are biologically interconnected. Epigenetic states can stabilize or prolong transcriptional responses, metabolic rewiring can alter redox balance and signaling activity, and autophagy-organelle quality control can determine whether mitochondrial stress, inflammasome activation, and other inflammatory signals are resolved or sustained. Their interaction ultimately shapes whether macrophages adopt inflammatory, clearance-related, resolution-associated, reparative, or tissue-supportive programs within a given disease niche. Genetic perturbation is considered separately as an experimental approach for causal interrogation of these regulatory systems rather than as an endogenous regulatory mechanism. From a functional perspective, macrophage reprogramming should likewise not be equated with suppression of inflammatory activation alone. Effective disease modification may also require restoration of resolution competence, including efferocytosis, debris clearance, lysosomal degradation, autophagic flux, pro-resolving mediator production, and repair-permissive communication. Accordingly, this section examines how disease-niche signals are decoded by interconnected macrophage regulatory systems and how these processes generate disease-relevant functional states.

### 3.1 Genetic perturbation and epigenetic control of macrophage state stability

Gene perturbation technologies, especially CRISPR/Cas9, have provided powerful tools for dissecting macrophage-regulatory genes and establishing causal mechanisms [124], [125], [126], [127]. For example, downregulation of HK2 using CRISPR/Cas9 can alter macrophage metabolic states and reshape inflammatory function [128]. Transcription activator-like effector nuclease (TALEN) approaches have also been applied to disease models involving macrophage dysfunction. In induced pluripotent stem cells (iPSCs) derived from patients, correction of GM-CSF receptor signaling can generate macrophages with restored phagocytic activity and cytokine secretory function [129]. These studies demonstrate the value of genetic perturbation for defining macrophage-regulatory mechanisms and establishing causal links between candidate genes and macrophage function.

Genetic perturbation technologies should therefore be clearly distinguished from clinically viable therapeutic strategies. CRISPR/Cas9, TALEN, and related loss- or gain-of-function approaches are primarily experimental tools for identifying macrophage-regulatory genes and establishing mechanistic or causal relationships. Their principal value lies in determining whether a candidate regulator is necessary or sufficient for macrophage dysfunction, rather than implying that genome editing itself represents an appropriate therapeutic strategy for chronic DMSDs. Direct therapeutic application of genome editing remains subject to substantial translational barriers, including unintended genomic effects, limited delivery efficiency and cell selectivity, ethical concerns, and uncertainties regarding long-term safety and treatment control. These limitations are particularly important in chronic, non-lethal disorders, for which the tolerance for irreversible treatment-related risk is relatively low. Accordingly, genetic perturbation should currently be regarded primarily as a platform for mechanistic validation and target discovery, while subsequent therapeutic development should prioritize interventions that are sufficiently controllable and compatible with long-term clinical use.

Epigenetic regulation offers a more dynamic explanation for macrophage plasticity. DNA methylation, RNA methylation, noncoding RNAs, and histone modifications can all regulate transcriptional programs associated with inflammation or inflammation resolution [130], [131], [132], [133], [134]. Factors such as DNMT3b, METTL3, HDAC5, and UTX influence macrophage function by modifying chromatin accessibility or RNA stability [125], [130], [133]. Noncoding RNAs, including miRNAs, can fine-tune macrophage states by regulating transcription factors, cytokine signaling, and metabolic enzymes [132]. The strength of epigenetic regulation lies in its reversibility and its capacity to shape cell states over time. It provides a useful framework for explaining how macrophages acquire inflammatory memory or reparative dysfunction within degenerative microenvironments. However, epigenetic interventions often affect broad transcriptional programs, and precise delivery systems targeting macrophage subsets within specific tissue niches remain insufficiently developed. Thus, epigenetic regulation is better regarded as a theoretical basis for understanding macrophage state stability and disease memory, rather than as a universal therapeutic strategy.

### 3.2 Metabolic control of inflammatory persistence and resolution

Macrophage function is tightly coupled to metabolic state. Pro-inflammatory macrophages often rely on enhanced glycolysis to support rapid adenosine triphosphate (ATP) production, inflammatory cytokine release, and reactive oxygen species (ROS) generation. By contrast, macrophages associated with inflammation resolution and tissue support are commonly linked to mitochondrial oxidative phosphorylation (OXPHOS), fatty acid oxidation, and metabolic homeostasis [135], [136], [137], [138], [139], [140], [141], [142]. Yet this metabolic distinction should not be treated as absolute, because macrophage metabolism in vivo is jointly shaped by nutrient availability, oxygen tension, mitochondrial quality, tissue pH, and inflammatory stimulation.

Within the microenvironment of DMSDs such as OA, OP, and IVDD, enhanced glycolysis, excessive ROS accumulation, and mitochondrial dysfunction can collectively sustain inflammatory macrophage programs. Current evidence suggests that suppressing glycolytic pathways, restoring mitochondrial metabolism, or eliminating excessive ROS can attenuate pro-inflammatory activation, thereby promoting inflammation resolution and tissue protection across different DMSD models [136], [137], [138], [139], [140], [143], [144], [145], [146], [147], [148], [149], [150], [151], [152], [153]. Inhibition of HIF-1α, GLUT1, ERK, or other drivers of glycolysis can attenuate inflammatory macrophage programs. At the same time, improving mitochondrial function, promoting fatty acid oxidation, or clearing excessive ROS may shift macrophages toward a less inflammatory state [138], [139], [140]. In OP and SP, immunometabolic regulation may also contribute to disease progression by influencing inflammaging, osteoclastogenesis, muscle mitochondrial function, and tissue repair.

Amino acid metabolism is another important node in macrophage regulation. Arginine can be metabolized by inducible nitric oxide synthase (iNOS) to produce nitric oxide (NO), thereby participating in inflammatory responses. Alternatively, it can be metabolized by Arg1 to generate ornithine and proline, which contribute to tissue repair and collagen metabolism [141]. Glutamine metabolism influences inflammatory and epigenetic states through the balance between α-ketoglutarate and succinate [142]. These metabolic nodes highlight that macrophage reprogramming is not controlled by a single signaling switch, but by coordinated regulation of metabolism, inflammation, and tissue repair.

Immunometabolic regulation reveals the coupling between inflammatory function and energy utilization, and provides a metabolic entry point for reversing unresolved inflammation. However, glycolysis, ROS, and mitochondrial function are not specific to macrophages. They are also essential for the homeostasis of chondrocytes, osteoblasts, NP cells, and myocytes. Therefore, the key to immunometabolic intervention is not systemic shutdown of a single metabolic pathway, but metabolic modulation adjusted to the local tissue context, disease stage, and relevant cell population.

### 3.3 Inflammatory signal decoding

NF-κB, MAPK, JAK/STAT, PI3K/Akt/mTOR, TGF-β, PPARγ, and related pathways jointly regulate macrophage cytokine production, survival, metabolism, and tissue repair functions [140], [143], [154], [155], [156], [157], [158], [159], [160]. NF-κB, MAPK, STAT1, IRF5, and HIF-1α are commonly associated with pro-inflammatory programs, whereas STAT3, STAT6, PPARγ, LXR, and IRF4 are more often involved in anti-inflammatory and tissue-supportive functions [157], [158], [159]. Many natural products, biomolecules, and biomaterials influence macrophage states through these signaling nodes.

Shared signaling pathways should not be regarded as disease-specific switches. Their biological effects are progressively shaped at several levels. First, niche-derived cues such as cytokines, hypoxia, nutrient availability, oxidative stress, and mechanical loading determine the threshold, intensity, and duration of pathway activation. Second, macrophage-intrinsic states modify how these signals are decoded: epigenetic priming can stabilize or prolong transcriptional responses, metabolic rewiring can alter redox balance and inflammatory signaling, and autophagy-mitophagy can determine whether mitochondrial stress and inflammasome activation are resolved or sustained. Third, the resulting macrophage outputs act on different resident-cell networks in each tissue. Consequently, similar inflammatory signaling may drive fibroblast activation and chondrocyte catabolism in OA, shift osteoclast-osteoblast coupling in OP, promote NP-cell dysfunction and matrix degradation after loss of disc immune restriction in IVDD, or support early debris clearance but impair regeneration when persistent in aging muscle. Thus, tissue-level outcome is determined not by pathway identity alone, but by pathway dynamics, macrophage intracellular state, spatial context, and the downstream cellular circuitry of each disease niche.

These shared signaling pathways are broadly utilized by multiple cell types within degenerative musculoskeletal tissues. Therefore, pathway inhibition or changes in macrophage marker expression should be interpreted together with functional macrophage readouts and disease-relevant tissue outcomes. A more reliable evaluation should connect macrophage signaling changes with endpoints that reflect the affected tissue, such as cartilage matrix preservation, improved bone remodeling balance, disc mechanical stability, or the quality of muscle regeneration.

### 3.4 Autophagy and organelle quality control

Compared with inflammatory signaling and immunometabolic rewiring, macrophage autophagy and organelle quality control represent a less extensively validated but biologically important therapeutic axis in DMSDs. Autophagy is a key quality-control mechanism in macrophages. Autophagy and mitophagy regulate macrophage function by clearing damaged organelles, reducing mitochondrial ROS, limiting inflammasome activation, maintaining lysosomal homeostasis, and supporting efferocytosis and tissue repair [161], [162], [163], [164], [165], [166]. Impaired autophagic flux can promote inflammatory activation, whereas appropriate restoration of autophagy may help restrain excessive inflammation and preserve macrophage repair-supporting capacity. Although autophagy and organelle stress are broadly relevant to macrophage biology across degenerative tissues, direct therapeutic examples that explicitly target macrophage autophagic or organelle quality-control programs are currently more limited and disease-specific. Macrophage autophagy and organelle quality control should therefore be viewed as context-dependent mechanisms for restoring cellular competence rather than as universally applicable therapeutic targets across DMSDs.

### 3.5 Niche-dependent regulation and evidence boundaries

Microenvironmental control represents the upstream foundation of macrophage plasticity. Oxygen tension, acidic pH, matrix stiffness, mechanical loading, cytokine gradients, and tissue damage signals can all shape macrophage function [167]. This explains why the same intervention may produce different outcomes in OA, OP, IVDD, and SP. Accordingly, disease-niche signals should be viewed as upstream determinants of how inflammatory signaling, metabolism, epigenetic state, and organelle quality control are integrated within macrophages. The resulting macrophage outputs then act on different resident-cell networks, producing disease-specific tissue outcomes. The synovial joint cavity, osteoporotic bone marrow, degenerative intervertebral disc, and aging muscle each possess distinct chemical and mechanical conditions. The advantage of biomaterials lies in their ability to locally shape macrophage behavior through surface chemistry, mechanical properties, drug release profiles, and lesion-responsive functions. Nevertheless, their long-term safety, degradability, and tissue compatibility require rigorous validation.

Because many macrophage-related pathways are also active in resident tissue cells, macrophage marker changes alone cannot establish macrophages as the main therapeutic target. Evidence for macrophage involvement should therefore be graded hierarchically. Marker conversion provides only associative evidence. Functional assays, including phagocytosis, efferocytosis, metabolic flux, ROS generation, autophagic flux, cytokine secretion, and macrophage-conditioned effects on resident tissue cells, provide stronger evidence of macrophage reprogramming. More convincing causal evidence requires macrophage depletion, blockade of monocyte recruitment, myeloid-specific genetic manipulation, macrophage-selective delivery, adoptive transfer, or rescue experiments. The strongest evidence links macrophage-dependent mechanisms to disease-relevant structural or functional improvement rather than to immune marker changes alone.

Together, these regulatory mechanisms should not be viewed as isolated molecular categories, but as context-dependent decoding systems that connect degenerative niche signals to macrophage functional output. Their biological meaning differs across DMSDs. This disease-specific interpretation provides the mechanistic basis for understanding how physical stimulation, natural compounds, biomaterials, extracellular vesicles (EVs), and gene-based tools regulate macrophage function in distinct pathological contexts, as summarized in Table 2.

**Table 2:** Mechanism-guided therapeutic applications and validation priorities for macrophage reprogramming in DMSDs.

Mechanistic axis	Therapeutic module	Core therapeutic logic	Representative DMSD examples	Key validation and translational considerations	Ref.
**Microenvironmental and niche-dependent regulation**	Niche-stress modulation	Reduce persistent mechanical, oxidative, metabolic, or inflammatory inputs that sustain maladaptive macrophage activation.	**OA:** exercise, LIPUS, HTO, and local antioxidant/lubricating platforms. **OP:** osteoimmune and ion-responsive bone-repair materials. **IVDD:** ROS-scavenging MnO_2_@TMNP and CeMn-PEG.	Demonstrate lesion-localized macrophage modulation together with disease-relevant tissue benefit. Delivery feasibility is highly niche dependent; macrophage-directed niche modulation in SP remains insufficiently established.	[147], [148], [149], [150], [151], [168], [169], [170], [171], [172], [173], [174], [175], [176], [177], [178], [179], [180]
**Inflammatory signaling pathways**	Inflammatory signal decoding	Redirect shared inflammatory signaling to limit persistent macrophage-mediated tissue injury without broadly suppressing physiological signaling.	**OA:** spermidine-mediated MAPK regulation. **OP:** bilobalide/SIRT3-NF-κB and Astragaloside IV/STING-NF-κB. **IVDD:** NF-κB-STAT3 and Zhiqiao Gancao decoction/JAK2-STAT3.	Pathway inhibition alone does not establish macrophage specificity. Validation should combine macrophage functional or causal evidence with spatial context and tissue-level rescue.	[181], [182], [183], [184], [185]
**Immunometabolic and redox regulation**	Metabolic and redox decoding	Restore macrophage metabolic and redox fitness by limiting glycolytic/ROS amplification and supporting mitochondrial homeostasis and resolution capacity.	**OA:** representative antioxidant and metabolic nanoplatforms. **IVDD:** iMSC-EVs that suppress pro-inflammatory glycolytic reprogramming within the macrophage-NP-cell circuit.	Confirm macrophage-specific changes in glycolysis, ROS, mitochondrial function, or OXPHOS and distinguish them from direct effects on resident tissue cells. Evidence remains predominantly preclinical.	[32], [153], [171], [186], [187], [188]
**Autophagy and organelle quality control**	Quality-control restoration	Restore autophagic flux, lysosomal function, mitophagy, mitochondrial quality, and inflammasome restraint to recover macrophage cellular competence.	**IVDD:** TMNP@SR-mediated regulation of macrophage autophagy. Aging muscle/SP-related models: LA-Au NPs enhancing lysosomal autophagy and mitochondrial OXPHOS.	Therapeutic relevance requires direct demonstration of macrophage quality-control restoration followed by disease-relevant structural or functional rescue. Excessive or nonspecific autophagy activation should be avoided.	[189], [190]

**Genetic perturbation and epigenetic control of macrophage state stability**	Causal validation and target discovery	Define macrophage-causal regulators and mechanisms governing state stability, inflammatory memory, and RNA/chromatin regulation.	**OA:** EV/miR-212-5p-NLRP3 m6A regulation. **IVDD:** DNMT1/SIRT6-related macrophage regulation and FCGR2A knockdown for causal validation.	Best positioned for mechanistic validation and target discovery. Macrophage-specific perturbation and tissue-level rescue strengthen causality, whereas permanent genome editing currently has limited direct translational applicability in chronic DMSDs.	[191], [192], [193]

Note: Communication-mediated repair is not listed as a separate macrophage-intrinsic mechanistic axis because it represents the downstream process through which macrophage reprogramming is translated into effects on resident cells and tissue outcomes; this process is discussed separately in Section 4.4. Abbreviations: DMSDs, degenerative musculoskeletal diseases; EVs, extracellular vesicles; HTO, high tibial osteotomy; iMSC, induced pluripotent stem cell-derived mesenchymal stem cell; IVDD, intervertebral disc degeneration; LIPUS, low-intensity pulsed ultrasound; NP, nucleus pulposus; OA, osteoarthritis; OP, osteoporosis; OXPHOS, oxidative phosphorylation; ROS, reactive oxygen species; SP, sarcopenia; MnO_2_@TMNP, MnO_2_ nanoparticles coated with TrkA-overexpressing macrophage membranes; CeMn-PEG, polyethylene glycol-capped cerium–manganese bimetallic nanozyme; TMNP@SR, TrkA-overexpressing macrophage-like SiO_2_–rapamycin nanoparticles; LA-Au NPs, lipoic acid-modified gold nanoparticles.

## 4 Therapeutic strategies for macrophage modulation across disease niches

Macrophage-directed interventions in DMSDs include physical stimulation, small molecules, biomolecules, EVs, nanomaterials, hydrogels, and selected nucleic-acid-based approaches. Rather than classifying these strategies by intervention format, we organize them according to therapeutic logic: reducing upstream niche stress, redirecting inflammatory and metabolic decoding, restoring macrophage quality-control capacity, and translating macrophage reprogramming into tissue-level repair. These interconnected modules link disease-niche inputs to macrophage competence, functional output, and tissue-level responses.

### 4.1 Reducing upstream niche stress to prevent maladaptive macrophage activation

In OA, upstream niche stress is generated by cartilage-derived debris, abnormal mechanical loading, inflammatory mediators, and oxidative stress within the synovial joint. These persistent inputs sustain maladaptive synovial macrophage activation and provide a rationale for interventions that modify the local joint environment [46], [194]. Moderate exercise has been reported to promote a macrophage profile associated with inflammatory resolution through PPARγ-related mechanisms [168]. Low-intensity pulsed ultrasound similarly reduces JNK and NF-κB signaling while increasing macrophage markers associated with repair-supportive responses [169]. Following high tibial osteotomy, correction of abnormal lower-limb alignment is accompanied by reduced synovial inflammation and a macrophage phenotypic shift characterized by decreased inflammatory markers and increased expression of markers classified as M2-associated in the original study [170]. These interventions are not macrophage drugs in a narrow sense, but they reduce the pathological inputs that instruct macrophage behavior. Antioxidant nanosystems, lubricating materials, and local delivery platforms further extend this logic by prolonging intra-articular exposure, scavenging ROS, improving lubrication, and reducing synovial inflammatory stress [147], [148], [171]. Their shared purpose is to convert the synovial joint from a stress-amplifying niche into a more repair-permissive environment.

In OP, niche stress is distributed across the bone marrow and bone-remodeling unit, where age-related, hormonal, mechanical, metabolic, and oxidative stress signals collectively disturb osteoimmune homeostasis [172], [173], [174]. Inorganic, metal-containing, and ion-responsive bone repair materials can reshape the osteoimmune repair niche through ion-associated signaling, material surface interactions, ROS regulation, inflammatory modulation, osteoconduction, and local structural support [175], [176], [177], [178]. A biomimetic bone glue composed of levodopa/oxidized chitosan and poly(lactic-co-glycolic acid) microspheres incorporating metal–organic framework-loaded nanohydroxyapatite (DOPM) further provides a local immune and structural environment for bone defect repair by regulating senescent macrophage states and inflammatory tone [179]. In this disease context, the goal of niche-stress modulation is not simply to suppress inflammation, but to restore a bone-forming and vascularized microenvironment in which macrophage-related signals no longer favor osteoclast dominance.

In IVDD, niche-stress modulation is constrained by the anatomy and mechanics of the intervertebral disc. The disc is avascular, nutrient limited, matrix dense, acidic, and mechanically confined. During degeneration, ROS accumulation, acidic pH, ECM disruption, endplate changes, and abnormal mechanical loading jointly injure NP cells and promote immune cell recruitment. MnO_2_@TMNP, coated with tropomyosin receptor kinase A (TrkA)-overexpressing macrophage membranes, scavenges ROS, suppresses inflammatory macrophage responses, and alleviates the inflammatory microenvironment and pain associated with disc degeneration [151]. A polyethylene glycol (PEG)-capped cerium–manganese bimetallic nanozyme (CeMn-PEG) scavenges ROS, promotes macrophage transition toward a resolution-associated state, and delays IVDD progression [180]. These strategies are relevant because oxidative stress acts as a common amplifier of NP cell injury, macrophage activation, matrix degradation, and pain-related inflammation. However, in IVDD, niche-stress modulation must also satisfy biomechanical requirements, including annulus fibrosus integrity, intradiscal pressure preservation, matrix retention, and mechanical stability.

In SP, niche-stress modulation remains less clearly developed than in OA, OP, and IVDD. This limitation reflects the nature of the disease niche itself. Aging muscle does not present as a localized inflammatory cavity, a bone defect microenvironment, or a confined matrix compartment that can be directly reshaped by local delivery. Instead, its macrophage-regulatory environment is dispersed across chronic low-grade inflammation, mitochondrial dysfunction, altered nutrient sensing, neuromuscular impairment, vascular insufficiency, ECM remodeling, and declining MuSC competence [36], [195], [196]. These systemic and tissue-wide stresses make macrophage-directed therapy more difficult in sarcopenia than localized intervention in the joint or intervertebral disc. Broad systemic suppression of macrophage activity may be problematic because macrophages contribute to tissue immune surveillance, inflammatory resolution, and physiological remodeling; experimental disruption of post-resolution macrophage function has been shown to impair local tissue immunity and promote tissue injury and fibrosis [197]. Future strategies should therefore favor transient and context-dependent modulation and, where feasible, combine muscle-enriched delivery with recognition of disease-associated macrophage states or local microenvironment-responsive activation, thereby limiting exposure of macrophages in non-target organs. Another important limitation is the heavy reliance on acute muscle-injury models. Sarcopenia develops within a chronic, age-associated systemic environment involving metabolic dysregulation, persistent low-grade inflammation, and impaired muscle homeostasis, as further supported by recent human and aged-mouse studies linking systemic metabolic alterations to macrophage-dependent regulation of muscle maintenance and regeneration [198]. Acute injury models should therefore be regarded primarily as models of regenerative macrophage biology rather than direct surrogates for chronic sarcopenia. Future therapeutic validation should prioritize naturally aged or other chronic age-associated models and incorporate muscle strength, endurance, regenerative capacity, and systemic immune safety as key translational endpoints.

The feasibility of upstream niche modulation therefore depends on whether macrophage-driving stress is locally accessible, embedded within a remodeling unit, mechanically constrained, or distributed across a systemic aging environment.

### 4.2 Targeting inflammatory and metabolic decoding in macrophages

Once niche stress is sensed, macrophages convert local cues into tissue-active outputs through interconnected inflammatory, metabolic, redox, and inflammasome pathways. Therapeutic strategies in this axis therefore aim to redirect how macrophages decode degenerative signals rather than simply suppress a single pathway.

In OA, therapeutic modulation of macrophage decoding aims to prevent persistent synovial stress from being converted into inflammatory outputs that reinforce cartilage catabolism. Spermidine illustrates this therapeutic logic by inhibiting p38/JNK/MAPK signaling and promoting resolution-associated synovial macrophage responses, with cartilage protection depending more on synovial macrophage regulation than on direct chondrocyte effects [149]. Quercetin-related NLRP3 modulation and dendrimer-based delivery systems further suggest that macrophage decoding can be redirected through control of oxidative stress, inflammasome activity, and mitochondrial homeostasis [181], [186]. Other local biomaterial and nanomedicine platforms similarly regulate the inflammatory and metabolic joint microenvironment, but should be interpreted as broader immune-niche interventions rather than direct evidence for a single macrophage-specific mechanism [32], [171], [187], [188].

In OP, macrophage signaling should be interpreted within the osteoimmune remodeling unit, where inflammatory restraint is therapeutically meaningful only when it is coupled to improved balance between bone resorption, osteogenesis, and bone-material integration. Bilobalide regulates macrophage inflammatory responses through SIRT3/NF-κB signaling and is accompanied by reduced RANKL-induced osteoclastogenesis and improved bone mineral density [182]. Astragaloside IV suppresses STING/NF-κB signaling, attenuates macrophage senescence and inflammatory activation, and creates a more favorable environment for the osteogenic differentiation of bone marrow mesenchymal stem cells (BMSCs) [183]. Urolithin B reduces inflammatory macrophage responses through VEGFR2 inhibition and improves implant stability in osteoporotic mice [184]. These examples indicate that macrophage-directed signaling modulation in OP is best positioned as an adjunctive osteoimmune strategy that helps recalibrate bone resorption, bone formation, and bone-material integration.

In IVDD, macrophage-directed signaling modulation must reduce inflammatory and metabolic outputs within a mechanically vulnerable disc environment. FCGR2A knockdown provides a relatively direct example of this axis by inhibiting inflammatory macrophage polarization, reducing NF-κB phosphorylation, promoting STAT3-related reparative responses, and improving NP cell status and annulus fibrosus integrity in IVDD models [193]. Zhiqiao Gancao decoction also reduces macrophage activation through JAK2/STAT3 signaling and improves NP cell viability in macrophage-conditioned settings [185]. The translational challenge is that reducing macrophage inflammatory signaling is not sufficient if the intervention introduces new injury to the disc. In IVDD, inflammatory and metabolic decoding must be modified while preserving matrix retention, intradiscal pressure, annulus fibrosus integrity, and mechanical stability.

In aging muscle and sarcopenia-related regenerative dysfunction, inflammatory and metabolic decoding should be framed as a problem of macrophage competence under chronic aging stress. Early inflammatory macrophage activity is required for debris clearance after muscle injury, but aged muscle is characterized by persistent inflammatory signaling, mitochondrial dysfunction, oxidative stress, and impaired acquisition of repair-supporting macrophage functions. Macrophage sterol regulatory element-binding protein 1 (SREBP1) provides a metabolic example, because SREBP1 deficiency disrupts macrophage metabolic function, impairs inflammatory resolution, and compromises skeletal muscle regeneration [199]. Macrophage-derived Selenoprotein P further suggests that antioxidant capacity is required for macrophages to acquire a restorative profile and support efficient muscle regeneration in aged muscle [200]. These studies should not be presented as established timing-based therapies for sarcopenia. Instead, they indicate that macrophage metabolic and antioxidant fitness may be necessary for restoring regenerative support in the aged muscle niche.

Across DMSDs, inflammatory and metabolic decoding provides a shared therapeutic entry point, but its biological meaning differs by tissue context. The same molecular targets may influence synovial inflammatory amplification, osteoimmune remodeling, macrophage-NP cell feedback, or macrophage competence in aged muscle depending on the niche in which they are manipulated. Because many of these pathways are also required for the homeostasis of neighboring resident cells, pathway modulation cannot by itself establish macrophage-specific therapeutic action. Its translational value therefore depends on linking macrophage-directed signaling or metabolic changes to disease-relevant tissue outcomes while minimizing unintended effects on non-target cells.

### 4.3 Restoring macrophage quality-control programs for tissue repair

Macrophage reprogramming does not depend only on inflammatory pathway activity. It also depends on whether macrophages retain the intracellular capacity to clear damaged organelles, restrain mitochondrial ROS, maintain lysosomal degradation, resolve inflammasome activation, and support efferocytosis. When these quality-control programs fail, macrophages may remain inflammatory not because they receive stronger stimuli, but because they have lost the cellular machinery needed to terminate stress responses. Autophagy, lysosomal function, mitophagy, mitochondrial fitness, and inflammasome restraint therefore constitute an emerging therapeutic axis for restoring macrophage competence.

This axis should not be forced equally across all DMSDs. Many interventions influence autophagy or mitochondrial function in tissue cells, but only a smaller subset directly implicates macrophage quality-control programs. In IVDD, TMNP@SR, a biomimetic nanoparticle consisting of a rapamycin-loaded mesoporous silica core coated with a TrkA-overexpressing macrophage membrane, regulates macrophage autophagy, thereby reducing NP cell apoptosis [189]. The importance of this strategy is not simply that autophagy is activated. Its significance lies in restoring a macrophage function that may interrupt the feedback between macrophage stress, inflammatory amplification, and NP cell injury. For disc therapy, however, this approach remains incomplete unless autophagy restoration is connected to disc height, matrix hydration, endplate status, and biomechanical preservation.

In aging muscle and sarcopenia-related models, quality-control restoration may be relevant because macrophage repair-supporting activity depends on lysosomal and mitochondrial fitness. Lipoic acid-modified gold nanoparticles (LA-Au NPs) have been reported to enhance lysosomal autophagy and mitochondrial OXPHOS in macrophages, promote repair-supporting macrophage responses, and improve skeletal muscle repair [190]. This example is valuable because it links macrophage organelle function to regenerative support, rather than treating macrophage polarization as a surface phenotype. However, it should be interpreted as early preclinical evidence in aging muscle or SP-related models, not as a mature sarcopenia therapy. Its broader implication is that macrophage failure in aged muscle may partly reflect loss of intracellular fitness, which limits the ability of macrophages to support myoblast proliferation, survival, and differentiation.

Thus, quality-control restoration should be understood as a strategy for recovering macrophage competence. Its therapeutic promise is strongest when restored autophagic flux, lysosomal activity, mitophagy, or mitochondrial homeostasis is demonstrated in macrophages and then linked to tissue repair. At the same time, this axis requires caution. Excessive or nonspecific activation of autophagy may interfere with macrophage survival, phagocytosis, or tissue homeostasis. The goal is not maximal autophagy, but restoration of the quality-control capacity required for resolution and repair.

### 4.4 Translating macrophage reprogramming into tissue-level repair signals

Macrophage reprogramming becomes therapeutically meaningful only when macrophages change the behavior of surrounding tissues. A macrophage state that appears favorable in isolation may have little disease relevance if it does not alter chondrocyte catabolism, osteoblast-osteoclast balance, NP cell stress, MuSC activity, angiogenesis, innervation, or matrix remodeling. Therefore, macrophage-mediated tissue communication is not another intracellular mechanism. It is the output layer through which macrophage modulation influences disease fate.

Reprogrammed macrophages communicate through cytokines, chemokines, EVs, lipid mediators, metabolites, matrix-regulatory enzymes, growth factors, and ligand-receptor interactions [47], [172], [194], [201], [202], [203], [204], [205]. These signals can either sustain degeneration or create a repair-permissive environment. In OA, vesicles released by inflammatory chondrocytes and exosomes derived from BMSCs can shift macrophage phenotypes toward profiles associated with inflammation resolution and alleviate OA [206], [207]. Small extracellular vesicles derived from BMSCs stimulated with an electromagnetic field (EMF) and ultrasmall superparamagnetic iron oxide (USPIO) particles (EMF-USPIO-sEVs) further show that EV-based approaches may simultaneously regulate macrophage polarization, anabolic responses, and cartilage regeneration [208]. The value of these strategies lies in whether they reshape joint-level communication rather than simply delivering anti-inflammatory cargo.

In OP, exosome-mediated signals from BMSCs, plasma, tissue vesicles, or exogenous biological vesicles can influence skeletal homeostasis by regulating bone formation, bone resorption, and local inflammatory tone [209], [210], [211], [212], [213]. BMSC-derived exosomes that upregulate TRIM25 and promote TREM1 degradation provide one example of how vesicle-mediated signaling can suppress excessive inflammatory macrophage activation [214]. However, in OP, communication-directed therapy should ultimately be judged by whether it supports osteogenesis, restrains osteoclast dominance, and improves vascularized bone repair.

In IVDD, reciprocal macrophage-NP-cell communication becomes therapeutically relevant as degeneration permits immune-cell entry and establishes inflammatory and metabolic feedback [215], [216], [217], [218]. EVs derived from induced pluripotent stem cell-derived mesenchymal stem cells (iMSCs) can improve senescent NP cell function and reshape macrophage phenotypes by inhibiting pro-inflammatory glycolytic reprogramming [153]. In this setting, communication-directed therapy should aim to interrupt the macrophage-NP inflammatory metabolic circuit while preserving disc matrix integrity and biomechanical stability.

In SP, communication-mediated repair is a more convincing therapeutic angle than direct timing-based macrophage therapy. Macrophages influence MuSC activation, myoblast proliferation and differentiation, angiogenesis, ECM remodeling, and myofiber repair through paracrine and ligand-receptor signals. IL-25 treatment and adoptive transfer of IL-25-induced macrophages have been reported to improve muscle mass and function in aged mice, partly through macrophage-derived Sonic Hedgehog signaling and downstream Akt/mTOR activation in muscle tissue [219]. Mesencephalic astrocyte-derived neurotrophic factor (MANF) provides another example of aging-impaired macrophage communication, because macrophage-derived MANF is required for efficient skeletal muscle regeneration and is reduced in aged regenerative contexts [220]. These findings support the idea that macrophage-directed therapy in SP should focus on restoring macrophage-MuSC and macrophage-myofiber communication, rather than assuming that continuous anti-inflammatory polarization is sufficient.

Across diseases, macrophage communication is the point at which immune modulation becomes tissue repair. This is why macrophage-targeted therapy cannot be assessed only by immune markers or pathway changes. Its final value depends on whether macrophages can redirect the local tissue conversation toward cartilage preservation, balanced bone remodeling, disc matrix stability, pain reduction, myofiber regeneration, or functional recovery.

### 4.5 Evaluating therapeutic translation in disease-specific contexts

The translational value of macrophage modulation depends on whether macrophages occupy a necessary and disease-relevant position in the therapeutic process. This point is particularly important because many interventions discussed above, including natural products, EVs, nanomaterials, hydrogels, scaffold materials, and physical approaches, act on multiple cell types simultaneously. Their benefits may result from antioxidant activity, mechanical support, lubrication, matrix protection, drug retention, vascular regulation, or direct effects on resident tissue cells. Therefore, improved disease outcomes should not automatically be attributed to macrophage reprogramming unless macrophage-dependent mechanisms are functionally demonstrated.

A more useful translational framework is to define what macrophage modulation contributes within each disease niche. OA offers a relatively accessible setting for local macrophage modulation, whereas in OP such approaches are more appropriately positioned as adjunctive osteoimmune strategies alongside established antiresorptive or osteoanabolic treatment. In IVDD, macrophage targeting has a strong biological rationale because inflammation and oxidative stress contribute to NP-cell injury, matrix degradation, and pain; however, local intradiscal delivery must be balanced against the structural injury caused by annular penetration. Repeated puncture, excessive injection volume, or high delivery pressure may compromise annulus fibrosus integrity, reduce intradiscal pressure, and potentially accelerate degeneration [221]. This mechanical burden may be reduced through low-volume, sustained-release strategies that limit repeated intervention. Alternative routes, including trans-endplate transport for suitable cargos and stage-dependent targeting of the peridiscal or neurovascular niche, may further reduce the need for direct penetration of the central disc [222], [223]. In SP, macrophage targeting remains less mature. Although aged muscle regeneration depends on macrophage-mediated debris clearance, inflammatory resolution, metabolic fitness, and repair-supporting communication, current evidence is still insufficient to define macrophage modulation as an established disease-modifying strategy for sarcopenia.

Therefore, macrophage-directed therapy in DMSDs should be advanced according to disease-specific feasibility, mechanistic necessity, and endpoint relevance. The strongest studies will not merely show changes in macrophage markers, but will demonstrate that macrophages are functionally required for therapeutic efficacy, that the intervention can safely reach the relevant tissue niche, and that macrophage modulation is linked to structural or functional outcomes that matter for each disease.

## 5 Challenges and future directions

Although macrophage plasticity provides an important immunological perspective for understanding DMSDs, its clinical translation still faces multiple challenges. First, the field has moved beyond the initial identification of macrophage heterogeneity, with multiple disease-associated states now described across OA, OP, IVDD, and aging muscle. The key challenge is therefore no longer simply to catalogue additional subsets, but to determine which populations are reproducible across patients and models, where they are located within diseased tissues, how they arise, and whether they are causally involved in degeneration or repair. Future studies should integrate single-cell or single-nucleus sequencing with spatial approaches, lineage or fate mapping where feasible, and functional perturbation. This is particularly important in IVDD, where macrophage abundance and localization vary with disease stage and anatomical disruption, whereas the developmental origin of human disc macrophages remains poorly resolved.

Second, therapeutic timing is a decisive determinant of efficacy. In SP, early inflammatory macrophages are responsible for clearing necrotic tissue, and premature intervention may disrupt the natural repair process. In IVDD, early stage discs may still retain a relatively intact immune barrier, whereas macrophage activity becomes more prominent after neurovascular ingrowth at later stages. In OA, inflammatory phenotypes and mechanically dominant phenotypes may respond differently to macrophage modulation. Future therapeutic strategies therefore need to move beyond uniform polarization control and toward stage-specific immunomodulation.

Third, macrophage phenotyping should be interpreted with methodological caution. Macrophage marker panels remain useful for describing experimentally induced states, but they cannot by themselves define macrophage identity, function, or macrophage-dependent disease modification. When studies employ conventional M1-or M2-polarizing conditions, these terms should be treated as descriptions of the experimental system rather than as evidence of discrete macrophage identities in diseased tissues. Future studies should therefore integrate marker expression with functional assays, spatial localization, cellular origin or lineage information where available, and disease-specific tissue outcomes. In OP, macrophages, osteoclast precursors, and mature osteoclasts belong to overlapping monocyte-macrophage lineage trajectories, making it necessary to distinguish osteoclastogenesis from macrophage reprogramming. In IVDD, the low abundance and uneven distribution of immune cells make macrophage detection sensitive to disease stage, sampling region, tissue digestion, and annulus fibrosus disruption. In SP, macrophage responses in acute injury models should not be directly equated with chronic aging-associated muscle degeneration.

Fourth, cell-type selectivity, delivery, safety, and clinical compatibility remain major translational bottlenecks. Many inflammatory and metabolic pathways targeted in macrophages, including NF-κB, MAPK, JAK/STAT, glycolysis, mitochondrial metabolism, and autophagy, are also required for the homeostasis of neighboring resident cells. Therefore, tissue accumulation or pathway modulation alone cannot establish macrophage-selective therapeutic action. Greater selectivity may be achieved through several complementary strategies. Receptor-directed ligands, peptides, or antibodies can promote preferential uptake by macrophages when the targeted receptor is sufficiently enriched in the relevant population; biomimetic carriers, including macrophage-membrane-coated nanoparticles and engineered extracellular vesicles, can enhance lesion homing and local retention; and particle size, surface properties, and phagocytic preference can be optimized to favor macrophage uptake. Local depots, hydrogels, and sustained-release systems can further reduce systemic exposure, while pH-, ROS-, or enzyme-responsive systems can restrict cargo release to pathological microenvironments [149], [151], [159], [189], [205]. These approaches generally provide preferential rather than absolute macrophage specificity, and their selectivity should therefore be verified by cell-resolved biodistribution and by confirming preservation of essential functions in neighboring resident cells. Disease-specific delivery constraints must also be considered: repeated intra-articular administration and long-term material retention are relevant in OA; bone-targeted systems in OP must remain compatible with osteogenesis and remodeling; intradiscal delivery in IVDD must preserve annulus fibrosus integrity and biomechanical stability; and systemic or distributed delivery in SP must avoid broad immune suppression [224].

Fifth, macrophage-targeted therapy must account for biological trade-offs rather than assuming that anti-inflammatory or repair-associated macrophage states are uniformly beneficial. Excessive suppression of inflammatory macrophages may impair debris clearance, apoptotic cell removal, host defense, and initiation of repair, whereas premature induction of repair-associated programs may be maladaptive if tissue debris, matrix damage, or necrotic fibers have not been adequately cleared. Broad inhibition of the monocyte-macrophage lineage may also interfere with physiological remodeling, and pro-repair signals may become harmful if they promote fibrosis, aberrant angiogenesis, neurovascular ingrowth, or mistimed regeneration. Future strategies should therefore aim for stage-specific immune recalibration rather than unidirectional macrophage polarization.

Looking forward, macrophage research in DMSDs should move from describing cell states to testing how macrophages influence tissue fate under clinically relevant conditions. Single-cell and spatial omics can identify niche-specific macrophage subsets and their interactions with chondrocytes, osteoblast-lineage cells, osteoclasts, NP cells, satellite cells, endothelial cells, and neural components. Functional assays can determine whether these macrophage states differ in phagocytosis, efferocytosis, metabolism, autophagic flux, cytokine output, inflammasome activation, or repair-supporting capacity. Biomaterials and delivery systems can improve lesion-localized modulation, but their value should be judged not only by material efficacy in animal models, but also by retention, degradation, immune compatibility, mechanical safety, dosing feasibility, and compatibility with standard therapies. These cross-cutting priorities are summarized in Fig. 2.

**Fig. 2:**
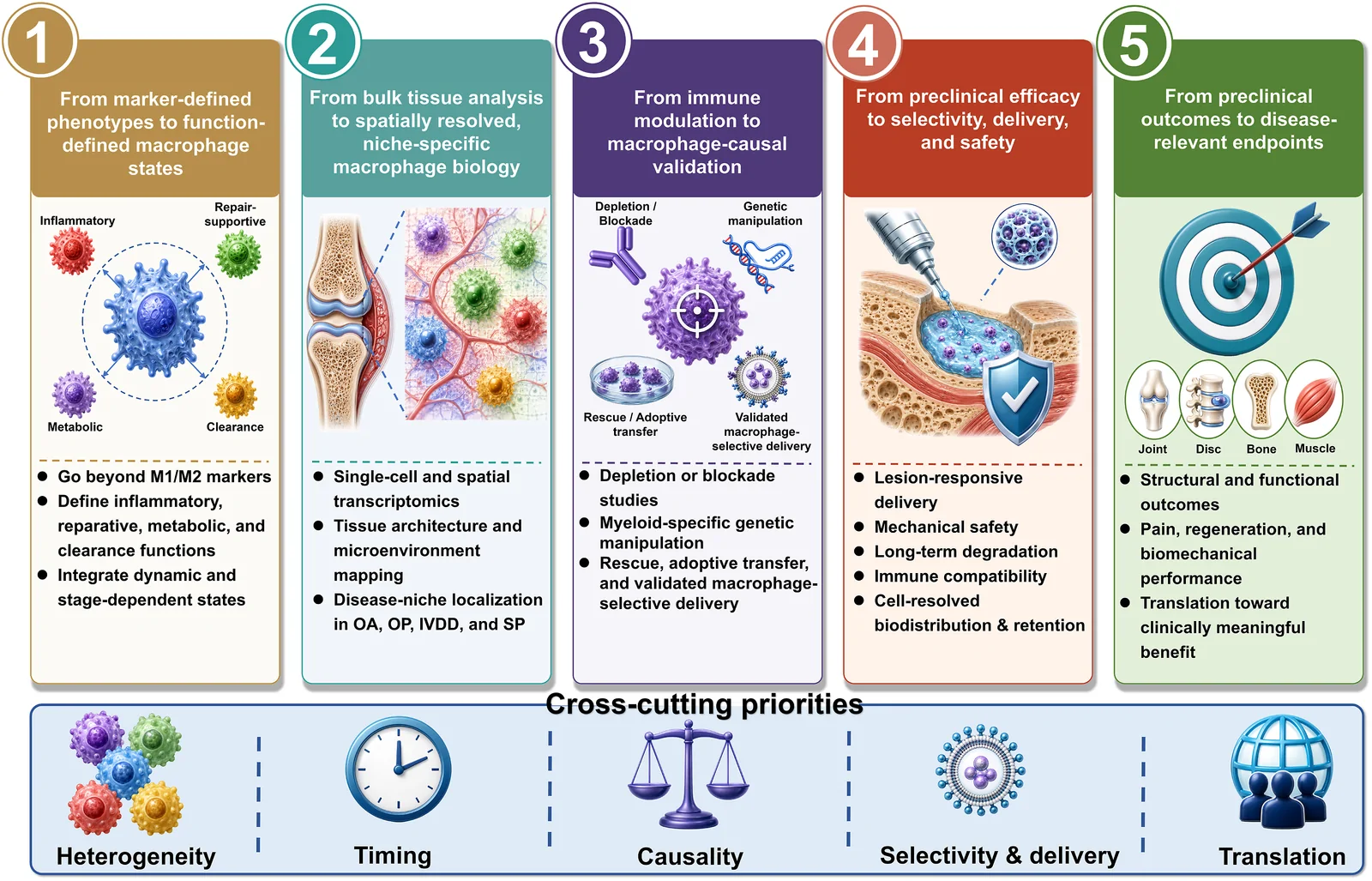
Future roadmap for macrophage-targeted therapy in degenerative musculoskeletal diseases. Future macrophage-targeted therapy in DMSDs should progress from marker-based phenotyping toward function-defined, niche-specific, and causally validated macrophage modulation. Key priorities include identifying disease-relevant macrophage subsets, defining stage-specific therapeutic windows, establishing macrophage-dependent mechanisms, improving cell-type selectivity, delivery safety, and tissue compatibility, and linking immune modulation to disease-specific structural and functional endpoints. Abbreviations: DMSDs, degenerative musculoskeletal diseases; IVDD, intervertebral disc degeneration; OA, osteoarthritis; OP, osteoporosis; SP, sarcopenia.

## 6 Conclusion

Macrophage reprogramming provides a useful framework for understanding DMSDs, but it should not be interpreted as a universal therapeutic route from inflammatory to anti-inflammatory phenotypes. Across degenerative musculoskeletal tissues, macrophages participate in inflammatory amplification, failed resolution, matrix remodeling, pain-related signaling, and insufficient repair. However, the meaning of these functions depends on the tissue niche in which macrophages operate, including local anatomy, mechanical loading, metabolic stress, immune accessibility, resident-cell composition, and reparative capacity.

This Review emphasizes that macrophage-directed therapy should be evaluated through a disease-niche-adapted logic. Therapeutic strategies may reduce upstream niche stress, redirect inflammatory and metabolic decoding, restore macrophage quality-control capacity, or reshape macrophage-mediated communication with resident tissue cells. Their value depends not on whether macrophage markers change, but on whether macrophages occupy a necessary position in the therapeutic effect and whether their modulation leads to tissue-relevant structural or functional improvement. The central translational question is therefore not whether an intervention can shift macrophage markers, but whether macrophage-dependent mechanisms are necessary for tissue-level protection and whether this protection can be achieved within the anatomical, mechanical, and delivery constraints of each disease niche.

Future studies should move beyond descriptive polarization markers and establish macrophage-causal evidence under disease-relevant conditions. By integrating tissue niche, regulatory mechanism, causal validation, delivery feasibility, and endpoint relevance, macrophage reprogramming may advance from a broad immunological concept toward precise and context-adapted therapeutic translation in degenerative musculoskeletal diseases.

## Author contributions

Chao Wang conceived the review, led the literature search, drafted the manuscript, and prepared the figures. Yuxin Chen contributed to the study design, literature search, figure preparation, and manuscript revision. Dong Ren, Yufeng Chen, Guowei Ren, and Tong Li contributed to literature collection and manuscript revision. Mehboob Ullah and Qin Xia provided conceptual advice and critical revision. Lei Dong and Pengcheng Wang supervised the work and revised the manuscript. All authors read and approved the final manuscript.

## Declaration of generative AI in scientific writing

No generative artificial intelligence (AI) or AI-assisted technologies were used in the preparation of this manuscript.

## Funding

This work was supported by the Beijing Municipal Natural Science Foundation (No. L2604023), the Hebei Province Major Science and Technology Support Program (No. 252W7702D), the General Program of the National Natural Science Foundation of China (NSFC) (No. 32570906), the Beijing Municipal Natural Science Foundation (No. L252006), the Beijing Municipal Natural Science Foundation (No. QY26238), the Fundamental Research Funds for the Central Universities, the Natural Science Foundation of Hebei Province (No. H2026206581), the Shandong Provincial Natural Science Foundation (Nos. ZR2024QC039, ZR2024MC035), and the Natural Science Foundation of Hebei Province (No. C2025105003).

## Declaration of competing interest

The author(s) have no conflicts of interest relevant to this article.

## References

[1] Cao F., Xu Z., Li X.X., Fu Z.Y., Han R.Y., Zhang J.L. (2024). Trends and cross-country inequalities in the global burden of osteoarthritis, 1990-2019: a population-based study. Ageing Res Rev.

[2] Perruccio A.V., Young J.J., Wilfong J.M., Denise Power J., Canizares M., Badley E.M. (2024). Osteoarthritis year in review 2023: epidemiology & therapy. Osteoarthr Cartil.

[3] Antoun E., Garratt E.S., Taddei A., Burton M.A., Barton S.J., Titcombe P. (2022). Epigenome-wide association study of sarcopenia: findings from the Hertfordshire Sarcopenia Study (HSS). J Cachexia Sarcopenia Muscle.

[4] Li B., Chen D. (2019). Degenerative musculoskeletal diseases: pathology and treatments. J Orthop Transl.

[5] Glyn-Jones S., Palmer A.J., Agricola R., Price A.J., Vincent T.L., Weinans H. (2015). Osteoarthritis. Lancet.

[6] Ensrud K.E., Crandall C.J. (2024). Osteoporosis. Ann Intern Med.

[7] Cieza A., Causey K., Kamenov K., Hanson S.W., Chatterji S., Vos T. (2021). Global estimates of the need for rehabilitation based on the global Burden of Disease study 2019: a systematic analysis for the global Burden of Disease Study 2019. Lancet.

[8] Sayer A.A., Cooper R., Arai H., Cawthon P.M., Ntsama Essomba M.J., Fielding R.A. (2024). Sarcopenia. Nat Rev Dis Primers.

[9] Yin P., Jiang Y., Fang X., Wang D., Li Y., Chen M. (2023). Cell-Based therapies for degenerative musculoskeletal diseases. Adv Sci (Weinh).

[10] Kloppenburg M., Namane M., Cicuttini F. (2025). Osteoarthritis. Lancet.

[11] Yue L., Berman J. (2022). What is osteoarthritis?. JAMA.

[12] Yao Q., Wu X., Tao C., Gong W., Chen M., Qu M. (2023). Osteoarthritis: pathogenic signaling pathways and therapeutic targets. Signal Transduct Targeted Ther.

[13] Wang H., Luo Y., Wang H., Li F., Yu F., Ye L. (2023). Mechanistic advances in osteoporosis and anti-osteoporosis therapies. MedComm (2020).

[14] Sözen T., Özışık L., Başaran N. (2017). An overview and management of osteoporosis. Eur J Rheumatol.

[15] Clynes M.A., Harvey N.C., Curtis E.M., Fuggle N.R., Dennison E.M., Cooper C. (2020). The epidemiology of osteoporosis. Br Med Bull.

[16] Hadji P., Klein S., Gothe H., Häussler B., Kless T., Schmidt T. (2013). The epidemiology of osteoporosis--Bone Evaluation Study (BEST): an analysis of routine health insurance data. Dtsch Arztebl Int.

[17] Silverstein W.K., Cantor N., Cheung A.M. (2024). Postmenopausal osteoporosis. N Engl J Med.

[18] Ye C., Morin S.N., Lix L.M., McCloskey E.V., Johansson H., Harvey N.C. (2024). Age at first fracture and later fracture risk in older adults undergoing osteoporosis assessment. JAMA Netw Open.

[19] Qadir A., Liang S., Wu Z., Chen Z., Hu L., Qian A. (2020). Senile osteoporosis: the involvement of differentiation and senescence of bone marrow stromal cells. Int J Mol Sci.

[20] Bisikirska B., Labella R., Cuesta-Dominguez A., Luo N., De Angelis J., Mosialou I. (2024). Melatonin receptor 1A variants as genetic cause of idiopathic osteoporosis. Sci Transl Med.

[21] Ebeling P.R., Nguyen H.H., Aleksova J., Vincent A.J., Wong P., Milat F. (2022). Secondary osteoporosis. Endocr Rev.

[22] Fu W., Chen M., Wang K., Chen Y., Cui Y., Xie Y. (2025). Tau is a receptor with low affinity for glucocorticoids and is required for glucocorticoid-induced bone loss. Cell Res.

[23] Lee E.J., Lialios P., Curtis M., Williams Jt, Kim Y., Salipante P. (2025). Glucocorticoids Alter bone microvascular barrier via MAPK/Connexin43 mechanisms. Adv Healthcare Mater.

[24] Ohnishi T., Iwasaki N., Sudo H. (2022). Causes of and molecular targets for the treatment of intervertebral disc degeneration: a review. Cells.

[25] Fine N., Lively S., Séguin C.A., Perruccio A.V., Kapoor M., Rampersaud R. (2023). Intervertebral disc degeneration and osteoarthritis: a common molecular disease spectrum. Nat Rev Rheumatol.

[26] Sun Z., Liu B., Luo Z.J. (2020). The immune privilege of the intervertebral disc: implications for intervertebral disc degeneration treatment. Int J Med Sci.

[27] Zheng B., Li S., Xiang Y., Zong W., Ma Q., Wang S. (2023). Netrin-1 mediates nerve innervation and angiogenesis leading to discogenic pain. J Orthop Transl.

[28] Wang X., Zhou L. (2023). The multifaceted role of macrophages in homeostatic and injured skeletal muscle. Front Immunol.

[29] Saini J., McPhee J.S., Al-Dabbagh S., Stewart C.E., Al-Shanti N. (2016). Regenerative function of immune system: modulation of muscle stem cells. Ageing Res Rev.

[30] Wen Z.Q., Lin J., Xie W.Q., Shan Y.H., Zhen G.H., Li Y.S. (2023). Insights into the underlying pathogenesis and therapeutic potential of endoplasmic reticulum stress in degenerative musculoskeletal diseases. Milit Med Res.

[31] Chen S., Saeed A., Liu Q., Jiang Q., Xu H., Xiao G.G. (2023). Macrophages in immunoregulation and therapeutics. Signal Transduct Targeted Ther.

[32] Wang F., Han X., Han Z., Wang J., Cai Z., Chen G. (2025). Slide-Ring structured stress-electric coupling Hydrogel microspheres for low-loss transduction between tissues. Adv Mater.

[33] Wang X., Liu C., Wang M., Yin B., Ge Y., Shu L. (2025). Multi-modal microcarriers reprogram mitochondrial metabolism and activate efferocytosis in macrophages for osteoporotic bone repair. Biomaterials.

[34] Zheng K., Wang S., Deng M., Luo Y., Li W., Zeng L. (2025). Mechanisms and therapeutic strategies of macrophage polarization in intervertebral disc degeneration. JOR Spine.

[35] Xu L., Qiu J., Ren Q., Wang D., Guo A., Wang L. (2025). Gold nanoparticles modulate macrophage polarization to promote skeletal muscle regeneration. Mater Today Bio.

[36] Cui C.Y., Ferrucci L., Gorospe M. (2023). Macrophage involvement in aging-associated skeletal muscle regeneration. Cells.

[37] Dou Y., Zhang Y., Liu Y., Sun X., Liu X., Li B. (2025). Role of macrophage in intervertebral disc degeneration. Bone Res.

[38] Hu K., Song M., Song T., Jia X., Song Y. (2025). Osteoimmunology in osteoarthritis: unraveling the interplay of immunity, inflammation, and joint degeneration. J Inflamm Res.

[39] Yang D.H., Yang M.Y. (2019). The role of macrophage in the pathogenesis of osteoporosis. Int J Mol Sci.

[40] Jakubzick C.V., Randolph G.J., Henson P.M. (2017). Monocyte differentiation and antigen-presenting functions. Nat Rev Immunol.

[41] Lazarov T., Juarez-Carreño S., Cox N., Geissmann F. (2023). Physiology and diseases of tissue-resident macrophages. Nature.

[42] Varol C., Mildner A., Jung S. (2015). Macrophages: development and tissue specialization. Annu Rev Immunol.

[43] Park M.D., Silvin A., Ginhoux F., Merad M. (2022). Macrophages in health and disease. Cell.

[44] Wynn T.A., Vannella K.M. (2016). Macrophages in tissue repair, regeneration, and fibrosis. Immunity.

[45] Li S., Yang Y., Qiu H., Pang L. (2026). Macrophage heterogeneity and plasticity: mechanism and therapeutics. Trends Pharmacol Sci.

[46] Liu Y., Duan J., Dang Y., Hao R., Wang H., Tan E. (2026). Remodeling of senescent macrophages in synovium alleviates trauma- and aging-induced osteoarthritis. Bioact Mater.

[47] Deng M., Jiang Y., Chen Z., Chen K., Cao N., Huang Y. (2026). Senescent synovial intimal fibroblasts aggravate osteoarthritis by regulating macrophage polarization and Chondrocyte phenotype through ANGPTL4-α5β1 axis. Adv Sci (Weinh).

[48] Kurowska-Stolarska M., Alivernini S. (2017). Synovial tissue macrophages: friend or foe?. RMD Open.

[49] Culemann S., Grüneboom A., Nicolás-Ávila J., Weidner D., Lämmle K.F., Rothe T. (2019). Locally renewing resident synovial macrophages provide a protective barrier for the joint. Nature.

[50] Xu M., Ji Y. (2023). Immunoregulation of synovial macrophages for the treatment of osteoarthritis. Open Life Sci.

[51] Lee Y.M., Kim M., Yuk H.J., Kim S.H., Kim D.S. (2023). Siraitia grosvenorii residual extract inhibits inflammation in RAW264.7 macrophages and Attenuates osteoarthritis progression in a rat model. Nutrients.

[52] Russo L., Lumeng C.N. (2018). Properties and functions of adipose tissue macrophages in obesity. Immunology.

[53] Kulkarni P., Srivastava V., Tootsi K., Electricwala A., Kharat A., Bhonde R. (2022). Synovial fluid in knee Osteoarthritis extends proinflammatory niche for macrophage polarization. Cells.

[54] Li G., Yin J., Gao J., Cheng T.S., Pavlos N.J., Zhang C. (2013). Subchondral bone in osteoarthritis: insight into risk factors and microstructural changes. Arthritis Res Ther.

[55] Utomo L., van Osch G.J., Bayon Y., Verhaar J.A., Bastiaansen-Jenniskens Y.M. (2016). Guiding synovial inflammation by macrophage phenotype modulation: an in vitro study towards a therapy for osteoarthritis. Osteoarthr Cartil.

[56] Geurts J., Patel A., Hirschmann M.T., Pagenstert G.I., Müller-Gerbl M., Valderrabano V. (2016). Elevated marrow inflammatory cells and osteoclasts in subchondral osteosclerosis in human knee osteoarthritis. J Orthop Res.

[57] Zhang W., Gao R., Rong X., Zhu S., Cui Y., Liu H. (2022). Immunoporosis: role of immune system in the pathophysiology of different types of osteoporosis. Front Endocrinol.

[58] Batoon L., Millard S.M., Raggatt L.J., Wu A.C., Kaur S., Sun L.W.H. (2021). Osteal macrophages support osteoclast-mediated resorption and contribute to bone pathology in a postmenopausal osteoporosis mouse model. J Bone Miner Res.

[59] Yao Y., Cai X., Ren F., Ye Y., Wang F., Zheng C. (2021). The macrophage-osteoclast axis in osteoimmunity and osteo-related diseases. Front Immunol.

[60] Luo G., Li F., Li X., Wang Z.G., Zhang B. (2018). TNF-α and RANKL promote osteoclastogenesis by upregulating RANK via the NF-κB pathway. Mol Med Rep.

[61] De Martinis M., Ginaldi L., Sirufo M.M., Pioggia G., Calapai G., Gangemi S. (2020). Alarmins in osteoporosis, RAGE, IL-1, and IL-33 pathways: a literature review. Medicina (Kaunas).

[62] Mun S.H., Park P.S.U., Park-Min K.H. (2020). The M-CSF receptor in osteoclasts and beyond. Exp Mol Med.

[63] Feng W., Liu H., Luo T., Liu D., Du J., Sun J. (2017). Combination of IL-6 and sIL-6R differentially regulate varying levels of RANKL-induced osteoclastogenesis through NF-κB, ERK and JNK signaling pathways. Sci Rep.

[64] Li X., Zhou Z.Y., Zhang Y.Y., Yang H.L. (2016). IL-6 contributes to the defective osteogenesis of bone marrow stromal cells from the vertebral body of the glucocorticoid-induced osteoporotic mouse. PLoS One.

[65] Muñoz J., Akhavan N.S., Mullins A.P., Arjmandi B.H. (2020). Macrophage polarization and osteoporosis: a review. Nutrients.

[66] Risbud M.V., Shapiro I.M. (2014). Role of cytokines in intervertebral disc degeneration: pain and disc content. Nat Rev Rheumatol.

[67] Nakazawa K.R., Walter B.A., Laudier D.M., Krishnamoorthy D., Mosley G.E., Spiller K.L. (2018). Accumulation and localization of macrophage phenotypes with human intervertebral disc degeneration. Spine J.

[68] Wang J., Zheng Z., Huang B., Wu H., Zhang X., Chen Y. (2020). Osteal tissue macrophages are involved in endplate osteosclerosis through the OSM-STAT3/YAP1 signaling axis in modic changes. J Immunol.

[69] Khan A.N., Jacobsen H.E., Khan J., Filippi C.G., Levine M., Lehman R.A. (2017). Inflammatory biomarkers of low back pain and disc degeneration: a review. Ann N Y Acad Sci.

[70] Kim C.F., Moalem-Taylor G. (2011). Detailed characterization of neuro-immune responses following neuropathic injury in mice. Brain Res.

[71] Ashraf S., Santerre P., Kandel R. (2021). Induced senescence of healthy nucleus pulposus cells is mediated by paracrine signaling from TNF-α-activated cells. FASEB J.

[72] Feng P., Che Y., Gao C., Zhu L., Gao J., Vo N.V. (2023). Immune exposure: how macrophages interact with the nucleus pulposus. Front Immunol.

[73] Wiedmer P., Jung T., Castro J.P., Pomatto L.C.D., Sun P.Y., Davies K.J.A. (2021). Sarcopenia - molecular mechanisms and open questions. Ageing Res Rev.

[74] Sun L., Wang X., Saredy J., Yuan Z., Yang X., Wang H. (2020). Innate-adaptive immunity interplay and redox regulation in immune response. Redox Biol.

[75] Krasniewski L.K., Chakraborty P., Cui C.Y., Mazan-Mamczarz K., Dunn C., Piao Y. (2022). Single-cell analysis of skeletal muscle macrophages reveals age-associated functional subpopulations. eLife.

[76] Tidball J.G. (2017). Regulation of muscle growth and regeneration by the immune system. Nat Rev Immunol.

[77] Murray P.J., Allen J.E., Biswas S.K., Fisher E.A., Gilroy D.W., Goerdt S. (2014). Macrophage activation and polarization: nomenclature and experimental guidelines. Immunity.

[78] Ginhoux F., Schultze J.L., Murray P.J., Ochando J., Biswas S.K. (2016). New insights into the multidimensional concept of macrophage ontogeny, activation and function. Nat Immunol.

[79] Wu L., Cao X., Shen B. (2025). Development of a macrophage polarization-modulating therapeutic agent for osteoarthritis treatment. J Orthop Surg Res.

[80] Liu C., Wang T., Ren Z., Luo J., Wang Z., Liang Y. (2026). The mechanism of action and therapeutic potential of macrophages in osteoporosis: from polarization balance to targeted regulation. Front Immunol.

[81] Hou Y., Zhao T., Guo Y., Shi J. (2025). Macrophage reprogramming as a therapeutic avenue in intervertebral disc degeneration: insights into pharmacological targeting. Biochem Biophys Res Commun.

[82] Moulin D., Sellam J., Berenbaum F., Guicheux J., Boutet M.A. (2025). The role of the immune system in osteoarthritis: mechanisms, challenges and future directions. Nat Rev Rheumatol.

[83] Lai Y., Ramírez-Pardo I., Isern J., An J., Perdiguero E., Serrano A.L. (2024). Multimodal cell atlas of the ageing human skeletal muscle. Nature.

[84] Li X., Fang L., Zhou R., Yao L., Clayton S.W., Muscat S. (2025). Current cutting-edge omics techniques on musculoskeletal tissues and diseases. Bone Res.

[85] Zhang H., Lin C., Zeng C., Wang Z., Wang H., Lu J. (2018). Synovial macrophage M1 polarisation exacerbates experimental osteoarthritis partially through R-spondin-2. Ann Rheum Dis.

[86] Yoneda T., Hiasa M., Nagata Y., Okui T., White F. (2015). Contribution of acidic extracellular microenvironment of cancer-colonized bone to bone pain. Biochim Biophys Acta.

[87] Laouteouet D., Bortolotti O., Marineche L., Hannemann N., Chambon M., Glasson Y. (2026). Monocyte-Derived macrophages-synovial Fibroblasts Crosstalk Unravels Oncostatin Signaling network as a driver of Synovitis in osteoarthritis. Arthritis Rheumatol.

[88] Raut R.D., Chakraborty A.K., Neogi T., Albro M., Snyder B., Schaer T.P. (2025). A multi-tissue human knee single-cell atlas identifies that osteoarthritis reduces regenerative tissue stem cells while increasing inflammatory pain macrophages. Commun Biol.

[89] Kulzhanova G., Klee A., Botros M., Hansen V.L., Reuter J., Tashbib E.T. (2026). Integrative single-cell RNA sequencing and spatial transcriptomics uncover distinct fibroblast-macrophage crosstalk in human hip synovium between patients with femoroacetabular impingement and osteoarthritis. Arthritis Rheumatol.

[90] Walker M.D., Shane E. (2023). Postmenopausal osteoporosis. N Engl J Med.

[91] Buckley L., Humphrey M.B. (2018). Glucocorticoid-Induced osteoporosis. N Engl J Med.

[92] Chen M., Lin W., Ye R., Yi J., Zhao Z. (2021). PPARβ/δ agonist alleviates diabetic osteoporosis via regulating M1/M2 macrophage polarization. Front Cell Dev Biol.

[93] Loi F., Córdova L.A., Zhang R., Pajarinen J., Lin T.H., Goodman S.B. (2016). The effects of immunomodulation by macrophage subsets on osteogenesis in vitro. Stem Cell Res Ther.

[94] Li L., Yu M., Li Y., Li Q., Yang H., Zheng M. (2021). Synergistic anti-inflammatory and osteogenic n-HA/resveratrol/chitosan composite microspheres for osteoporotic bone regeneration. Bioact Mater.

[95] Yang W., Wang Y., Mo K., Chen W., Xie X. (2024). Single-cell RNA sequencing reveals multiple immune cell subpopulations promote the formation of abnormal bone microenvironment in osteoporosis. Sci Rep.

[96] Bellido T. (2024). Bisphosphonates for osteoporosis: from bench to clinic. J Clin Investig.

[97] Kahwati L.C., Kistler C.E., Booth G., Sathe N., Gordon R.D., Okah E. (2025). Screening for osteoporosis to prevent fractures: a systematic evidence review for the US preventive services task force. JAMA.

[98] Masuda S., Fukasawa T., Matsuda S., Kawakami K. (2025). Cardiovascular safety and fracture prevention effectiveness of denosumab versus oral bisphosphonates in patients receiving dialysis : a target trial emulation. Ann Intern Med.

[99] Bird S.T., Gelperin K., Smith E.R., Jung T.H., Lyu H., Thompson A. (2025). The effect of denosumab on risk for emergently treated hypocalcemia by stage of chronic kidney disease : a target trial emulation. Ann Intern Med.

[100] Chen J.S., Sambrook P.N. (2011). Antiresorptive therapies for osteoporosis: a clinical overview. Nat Rev Endocrinol.

[101] Lu Y., Liu S., Yang P., Kou Y., Li C., Liu H. (2022). Exendin-4 and eldecalcitol synergistically promote osteogenic differentiation of bone marrow mesenchymal stem cells through M2 macrophages polarization via PI3K/AKT pathway. Stem Cell Res Ther.

[102] Li Z., Zhu X., Xu R., Wang Y., Hu R., Xu W. (2019). Deacylcynaropicrin inhibits RANKL-Induced osteoclastogenesis by inhibiting NF-κB and MAPK and promoting M2 polarization of macrophages. Front Pharmacol.

[103] Liu J., Li F., Ouyang Y., Su Z., Chen D., Liang Z. (2024). Naringin-induced M2 macrophage polarization facilitates osteogenesis of BMSCs and improves cranial bone defect healing in rat. Arch Biochem Biophys.

[104] Koroth J., Buko E.O., Abbott R., Johnson C.P., Ogle B.M., Stone L.S. (2023). Macrophages and intervertebral disc degeneration. Int J Mol Sci.

[105] Wang D., Li Z., Huang W., Cao S., Xie L., Chen Y. (2023). Single-cell transcriptomics reveals heterogeneity and intercellular crosstalk in human intervertebral disc degeneration. iScience.

[106] Jiang J., Kong F., Zheng B., Mei Z., Zhu J., Xu X. (2026). GPNMB(+) macrophages promote osteogenic differentiation of nucleus pulposus cells through PDGF signaling in intervertebral disc degeneration. Cell Rep Med.

[107] Liang G., Tan J., Chen C., Liu Y., Ye Y., Pan X. (2025). Single nuclear-spatial transcriptomic sequencing reveals distinct puncture-induced cell subpopulations in the intervertebral disc of a rat model. Clin Transl Med.

[108] Zhang K., Du L., Li Z., Huo Z., Shen L., Gao S. (2024). M2 macrophage-derived small extracellular vesicles ameliorate pyroptosis and intervertebral disc degeneration. Biomater Res.

[109] Nunes-Pinto M., Bandeira de Mello R.G., Pinto M.N., Moro C., Vellas B., Martinez L.O. (2025). Sarcopenia and the biological determinants of aging: a narrative review from a geroscience perspective. Ageing Res Rev.

[110] Kadi F., Ponsot E. (2010). The biology of satellite cells and telomeres in human skeletal muscle: effects of aging and physical activity. Scand J Med Sci Sports.

[111] Santoro A., Bientinesi E., Monti D. (2021). Immunosenescence and inflammaging in the aging process: age-related diseases or longevity?. Ageing Res Rev.

[112] Wang T. (2022). Searching for the link between inflammaging and sarcopenia. Ageing Res Rev.

[113] Wang Y., Welc S.S., Wehling-Henricks M., Tidball J.G. (2018). Myeloid cell-derived tumor necrosis factor-alpha promotes sarcopenia and regulates muscle cell fusion with aging muscle fibers. Aging Cell.

[114] Tylutka A., Walas Ł., Zembron-Lacny A. (2024). Level of IL-6, TNF, and IL-1β and age-related diseases: a systematic review and meta-analysis. Front Immunol.

[115] Ding J., Yang G., Sun W., Li Y., Wang N., Wang J. (2024). Association of interleukin-6 with sarcopenia and its components in older adults: a systematic review and meta-analysis of cross-sectional studies. Ann Med.

[116] Shen L., Zong Y., Zhao J., Yang Y., Li L., Li N. (2024). Characterizing the skeletal muscle immune microenvironment for sarcopenia: insights from transcriptome analysis and histological validation. Front Immunol.

[117] Fuchs E., Blau H.M. (2020). Tissue stem cells: architects of their niches. Cell Stem Cell.

[118] Cui C.Y., Ferrucci L. (2020). Macrophages in skeletal muscle aging. Aging (Albany NY).

[119] Forcina L., Cosentino M., Musarò A. (2020). Mechanisms regulating muscle regeneration: insights into the interrelated and time-dependent phases of tissue healing. Cells.

[120] Huang X., Lan Y., Shen J., Zhao X., Zhou Y., Wu W. (2024). M2 macrophages secrete glutamate-containing extracellular vesicles to alleviate osteoporosis by reshaping osteoclast precursor fate. Mol Ther.

[121] Gu Y., Wang K., Wang Y., Wang Z., Li Y., Li L. (2026). Macrophage metabolic reprogramming via HIF-1α-glycolysis drives osteoblast ferroptosis and bone loss through an IL-6-STAT3-dependent redox axis. Redox Rep.

[122] Fan C., Wang W., Yu Z., Wang J., Xu W., Ji Z. (2024). M1 macrophage-derived exosomes promote intervertebral disc degeneration by enhancing nucleus pulposus cell senescence through LCN2/NF-κB signaling axis. J Nanobiotechnol.

[123] Brorson J., Lin L., Wang J., Bæk A., Billeskov T.B., Thybo F.F. (2025). Complementing muscle regeneration-fibro-adipogenic progenitor and macrophage-mediated repair of elderly human skeletal muscle. Nat Commun.

[124] Ito Y., Inoue S., Kagoya Y. (2024). Gene editing technology to improve antitumor T-cell functions in adoptive immunotherapy. Inflamm Regen.

[125] Allemailem K.S., Alsahli M.A., Almatroudi A., Alrumaihi F., Al Abdulmonem W., Moawad A.A. (2023). Innovative strategies of reprogramming immune System cells by targeting CRISPR/Cas9-Based genome-editing tools: a new era of cancer management. Int J Nanomed.

[126] Poltavets A.S., Vishnyakova P.A., Elchaninov A.V., Sukhikh G.T., Fatkhudinov T.K. (2020). Macrophage modification strategies for efficient cell therapy. Cells.

[127] Tian X., Xie G., Xiao H., Ding F., Bao W., Zhang M. (2019). CXCR4 knockdown prevents inflammatory cytokine expression in macrophages by suppressing activation of MAPK and NF-κB signaling pathways. Cell Biosci.

[128] Huang W., Fu G., Wang Y., Chen C., Luo Y., Yan Q. (2024). Immunometabolic reprogramming of macrophages with inhalable CRISPR/Cas9 nanotherapeutics for acute lung injury intervention. Acta Biomater.

[129] Kuhn A., Ackermann M., Mussolino C., Cathomen T., Lachmann N., Moritz T. (2017). TALEN-mediated functional correction of human iPSC-derived macrophages in context of hereditary pulmonary alveolar proteinosis. Sci Rep.

[130] Lei H., He M., He X., Li G., Wang Y., Gao Y. (2021). METTL3 induces bone marrow mesenchymal stem cells osteogenic differentiation and migration through facilitating M1 macrophage differentiation. Am J Transl Res.

[131] Liu Y., Liu Z., Tang H., Shen Y., Gong Z., Xie N. (2019). The N(6)-methyladenosine (m(6)A)-forming enzyme METTL3 facilitates M1 macrophage polarization through the methylation of STAT1 mRNA. Am J Physiol Cell Physiol.

[132] Essandoh K., Li Y., Huo J., Fan G.C. (2016). MiRNA-Mediated macrophage polarization and its potential role in the regulation of inflammatory response. Shock.

[133] Peng W., Xie Y., Luo Z., Liu Y., Xu J., Li C. (2023). UTX deletion promotes M2 macrophage polarization by epigenetically regulating endothelial cell-macrophage crosstalk after spinal cord injury. J Nanobiotechnol.

[134] Sun D., Chang Q., Lu F. (2024). Immunomodulation in diabetic wounds healing: the intersection of macrophage reprogramming and immunotherapeutic hydrogels. J Tissue Eng.

[135] Wang Z., Zhao F., Xu C., Zhang Q., Ren H., Huang X. (2024). Metabolic reprogramming in skin wound healing. Burns Trauma.

[136] Zeng W., Li F., Jin S., Ho P.C., Liu P.S., Xie X. (2023). Functional polarization of tumor-associated macrophages dictated by metabolic reprogramming. J Exp Clin Cancer Res.

[137] Kim Y.J., Lee S., Jin J., Woo H., Choi Y.K., Park K.G. (2022). Cassiaside C inhibits M1 polarization of macrophages by downregulating glycolysis. Int J Mol Sci.

[138] Chang L., Gao J., Yu Y., Liao B., Zhou Y., Zhang J. (2023). MMP10 alleviates non-alcoholic steatohepatitis by regulating macrophage M2 polarization. Int Immunopharmacol.

[139] Zhang L., Chen X., Cai P., Sun H., Shen S., Guo B. (2022). Reprogramming mitochondrial metabolism in synovial macrophages of early osteoarthritis by a camouflaged meta-defensome. Adv Mater.

[140] Yuan Z., Jiang D., Yang M., Tao J., Hu X., Yang X. (2024). Emerging roles of macrophage polarization in osteoarthritis: mechanisms and therapeutic strategies. Orthop Surg.

[141] Guo C., Islam R., Zhang S., Fang J. (2021). Metabolic reprogramming of macrophages and its involvement in inflammatory diseases. EXCLI J.

[142] Liu P.S., Wang H., Li X., Chao T., Teav T., Christen S. (2017). α-ketoglutarate orchestrates macrophage activation through metabolic and epigenetic reprogramming. Nat Immunol.

[143] Yan L., Wang J., Cai X., Liou Y.C., Shen H.M., Hao J. (2020). Macrophage plasticity: signaling pathways, tissue repair, and regeneration. MedComm.

[144] Wu X., Xia Y., Dai H., Hong C., Zhao Y., Wei W. (2024). Metabolic control during macrophage polarization by a citrate-functionalized scaffold for maintaining bone homeostasis. Adv Healthcare Mater.

[145] Jia N., Gao Y., Li M., Liang Y., Li Y., Lin Y. (2023). Metabolic reprogramming of proinflammatory macrophages by target delivered roburic acid effectively ameliorates rheumatoid arthritis symptoms. Signal Transduct Targeted Ther.

[146] He X.X., Huang Y.J., Hu C.L., Xu Q.Q., Wei Q.J. (2024). Songorine modulates macrophage polarization and metabolic reprogramming to alleviate inflammation in osteoarthritis. Front Immunol.

[147] Wang H., Li Z., Liu J., Chang H., Wang J., Song S. (2024). Nanozyme-enhanced injectable hyaluronic acid-based hydrogel for the treatment of osteoarthritis. Int J Biol Macromol.

[148] Chen Z., Pang Q., Zhan J., Liu J., Zhao W., Dong L. (2024). MSCs-derived ECM functionalized hydrogel regulates macrophage reprogramming for osteoarthritis treatment by improving mitochondrial function and energy metabolism. Mater Today Bio.

[149] Deng C., Xiao Y., Zhao X., Li H., Chen Y., Ai K. (2025). Sequential targeting chondroitin sulfate-bilirubin nanomedicine attenuates Osteoarthritis via reprogramming lipid metabolism in M1 macrophages. Adv Sci (Weinh).

[150] Huang L., Wang J., Yu J., Bian M., Xiang X., Han G. (2024). Picein alleviates oxidative stress and promotes bone regeneration in osteoporotic bone defect by inhibiting ferroptosis via Nrf2/HO-1/GPX4 pathway. Environ Toxicol.

[151] Yang W., Li K., Pan Q., Huang W., Xiao Y., Lin H. (2024). An engineered bionic nanoparticle sponge as a cytokine trap and reactive oxygen species scavenger to relieve disc degeneration and discogenic pain. ACS Nano.

[152] Liu Y., Zhou K., He X., Shi K., Hu D., Yang C. (2025). A thermo-sensitive hydrogel targeting macrophage reprogramming for sustained osteoarthritis pain relief. Acta Pharm Sin B.

[153] Xiang Q., Zhan J., Tian S., Zhao Y., Wu Z., Lin J. (2025). Human iPSCs derived MSCs-Secreted exosomes modulate senescent Nucleus Pulposus cells induced macrophage polarization via metabolic reprogramming to mitigate intervertebral disc degeneration. Adv Sci (Weinh).

[154] Yang X., Qian H., Meng J., Jiang H., Yuan T., Yang S. (2023). Lonicerin alleviates the progression of experimental rheumatoid arthritis by downregulating M1 macrophages through the NF-κB signaling pathway. Phytother Res.

[155] Mandula J.K., Sierra-Mondragon R.A., Jimenez R.V., Chang D., Mohamed E., Chang S. (2024). Jagged2 targeting in lung cancer activates anti-tumor immunity via Notch-induced functional reprogramming of tumor-associated macrophages. Immunity.

[156] Wang W., Chu Y., Zhang P., Liang Z., Fan Z., Guo X. (2023). Targeting macrophage polarization as a promising therapeutic strategy for the treatment of osteoarthritis. Int Immunopharmacol.

[157] Lawrence T., Natoli G. (2011). Transcriptional regulation of macrophage polarization: enabling diversity with identity. Nat Rev Immunol.

[158] Xue J., Schmidt S.V., Sander J., Draffehn A., Krebs W., Quester I. (2014). Transcriptome-based network analysis reveals a spectrum model of human macrophage activation. Immunity.

[159] Li T., Li Q., Liu S., Cao J., Mei J., Gong J. (2025). Targeted V-type peptide-decorated nanoparticles prevent colitis by inhibiting endosomal TLR signaling and modulating intestinal macrophage polarization. Biomaterials.

[160] Chen Y.Q., Shao Y.C., Wei R.L. (2024). Pioglitazone alleviates lacrimal gland impairments induced by high-fat diet by suppressing M1 polarization. J Lipid Res.

[161] Wu M.Y., Lu J.H. (2019). Autophagy and macrophage functions: inflammatory response and phagocytosis. Cells.

[162] Liu K., Zhao E., Ilyas G., Lalazar G., Lin Y., Haseeb M. (2015). Impaired macrophage autophagy increases the immune response in obese mice by promoting proinflammatory macrophage polarization. Autophagy.

[163] Liu T., Wang L., Liang P., Wang X., Liu Y., Cai J. (2021). USP19 suppresses inflammation and promotes M2-like macrophage polarization by manipulating NLRP3 function via autophagy. Cell Mol Immunol.

[164] Zhou S., Gu J., Liu R., Wei S., Wang Q., Shen H. (2018). Spermine alleviates acute liver injury by inhibiting liver-resident macrophage pro-inflammatory response through ATG5-Dependent autophagy. Front Immunol.

[165] Luo Y., Lu S., Gao Y., Yang K., Wu D., Xu X. (2020). Araloside C attenuates atherosclerosis by modulating macrophage polarization via Sirt1-mediated autophagy. Aging (Albany NY).

[166] Kawano A., Ariyoshi W., Yoshioka Y., Hikiji H., Nishihara T., Okinaga T. (2019). Docosahexaenoic acid enhances M2 macrophage polarization via the p38 signaling pathway and autophagy. J Cell Biochem.

[167] Ke X., Chen C., Song Y., Cai Q., Li J., Tang Y. (2019). Hypoxia modifies the polarization of macrophages and their inflammatory microenvironment, and inhibits malignant behavior in cancer cells. Oncol Lett.

[168] Silveira L.S., Batatinha H.A.P., Castoldi A., Câmara N.O.S., Festuccia W.T., Souza C.O. (2019). Exercise rescues the immune response fine-tuned impaired by peroxisome proliferator-activated receptors γ deletion in macrophages. J Cell Physiol.

[169] Xu Z., Li S., Wan L., Hu J., Lu H., Zhang T. (2023). Role of low-intensity pulsed ultrasound in regulating macrophage polarization to accelerate tendon-bone interface repair. J Orthop Res.

[170] Yoshida S., Nishitani K., Yoshitomi H., Kuriyama S., Nakamura S., Fujii T. (2023). Knee alignment correction by high tibial osteotomy reduces symptoms and synovial inflammation in knee Osteoarthritis accompanied by macrophage phenotypic change from M1 to M2. Arthritis Rheumatol.

[171] Liu Y., Ma Z., Wang X., Liang J., Zhao L., Zhang Y. (2024). A core-brush nanoplatform with enhanced lubrication and anti-inflammatory properties for Osteoarthritis treatment. Adv Sci (Weinh).

[172] Wang D., Zhong D., He Y., Xie J., Ruan D., Liu L. (2026). Intercellular communication-driven mitochondrial transfer via CXCR4-Engineered macrophages reprograms bone marrow metabolism for osteoporosis therapy. Adv Mater.

[173] Zhu S., Yan M.Q., Masson A., Chen W., Li Y.P. (2026). Cell signaling and transcriptional regulation of osteoclast lineage commitment, differentiation, bone resorption and diseases. Cell Discov.

[174] Jiang X., Chen H., Hou W., Dai C., Zhang R., Chen E. (2026). Osteoblast-derived osteomodulin restrains osteoclastogenesis via ITGB8/RRM2-mediated reduction of mitochondrial respiration and mitochondrial ATP production. Exp Mol Med.

[175] Ji H., Shen G., Liu H., Liu Y., Qian J., Wan G. (2024). Biodegradable Zn-2Cu-0.5Zr alloy promotes the bone repair of senile osteoporotic fractures via the immune-modulation of macrophages. Bioact Mater.

[176] Xu L., Fang J., Pan J., Qi H., Yin Y., He Y. (2024). Zinc finger-inspired peptide-metal-phenolic nanointerface enhances bone-implant integration under bacterial infection microenvironment through immune modulation and osteogenesis promotion. Bioact Mater.

[177] Zhou H., He Z., Cao Y., Chu L., Liang B., Yu K. (2024). An injectable magnesium-loaded hydrogel releases hydrogen to promote osteoporotic bone repair via ROS scavenging and immunomodulation. Theranostics.

[178] Dai Q., Wang Z., Liu C., Chen X., Cao X. (2024). High performance injectable Mg doped bioactive glass bone cement for the regulation of osteogenic immune microenvironment. Biomater Adv.

[179] Li C., Xu W., Li L., Zhou Y., Yao G., Chen G. (2024). Concrete-Inspired bionic bone glue repairs osteoporotic bone defects by gluing and remodeling aging macrophages. Adv Sci (Weinh).

[180] Wu J., Chen Z., Huang H., Wang H., Wang X., Lu Z. (2024). Custom-Made Ce-Mn bimetallic nanozyme for the treatment of intervertebral disc degeneration by inhibiting oxidative stress and modulating Macrophage M1/M2 polarization. Biomater Res.

[181] Li W., He H., Du M., Gao M., Sun Q., Wang Y. (2024). Quercetin as a promising intervention for rat osteoarthritis by decreasing M1-polarized macrophages via blocking the TRPV1-mediated P2X7/NLRP3 signaling pathway. Phytother Res.

[182] Qin Y., Hu C., Jin J., Chao Y., Wang D., Xia F. (2024). Bilobalide ameliorates osteoporosis by influencing the SIRT3/NF-κB axis in osteoclasts and promoting M2 polarization in macrophages. Int J Biol Macromol.

[183] Li M., Niu Y., Tian L., Zhang T., Zhou S., Wang L. (2024). Astragaloside IV alleviates macrophage senescence and d-galactose-induced bone loss in mice through STING/NF-κB pathway. Int Immunopharmacol.

[184] Xiao L., Yang Y., Yu J., Li Y., Chen S., Gu Y. (2024). Urolithin B inhibits the differentiation of M1 macrophages and relieves the inflammation around the implants under osteoporosis via down-regulating the phosphorylation of VEGFR2. Int Immunopharmacol.

[185] Huang Z., Shen X., Chen H., Zhu Z., Lu B., Zhang L. (2024). Zhiqiao Gancao decoction regulated JAK2/STAT3/macrophage M1 polarization to ameliorate intervertebral disc degeneration. Heliyon.

[186] Sun H., Zhan M., Zou Y., Ma J., Liang J., Tang G. (2025). Bioactive phosphorus dendrimers deliver protein/drug to tackle osteoarthritis via cooperative macrophage reprogramming. Biomaterials.

[187] Cui S.H., Yan Y., Lu A., Dou Y., Li Z.W., Zhu Z.H. (2024). Nanomedicines promote cartilage regeneration in osteoarthritis by synergistically enhancing chondrogenesis of mesenchymal stem cells and regulating inflammatory environment. ACS Nano.

[188] Sun Y., Ding S.L., Zhao X., Sun D., Yang Y., Chen M. (2024). Self-Reinforced MOF-Based Nanogel Alleviates osteoarthritis by long-acting drug release. Adv Mater.

[189] Li K., Yang W., Chen X., Yu Y., Liu Y., Ni F. (2024). A structured biomimetic nanoparticle as inflammatory factor sponge and autophagy-regulatory agent against intervertebral disc degeneration and discogenic pain. J Nanobiotechnol.

[190] Xu L., Zhang Y., Wang D., Ren Q., Wang Y., Zang Z. (2025). Nanoparticle-Driven skeletal muscle repair and regeneration through macrophage-muscle stem cell interaction. Small.

[191] Shao X., Zhang M., Hu S., Yang Z. (2026). Extracellular vesicles from glucocorticoids-preconditioned synovial mesenchymal stem cells exert antiarthritic effects by downregulating the mRNA m6A modification of NLRP3 in macrophages through miR-212-5p. Bioeng Transl Med.

[192] Hou Y., Shi J., Guo Y., Shi G. (2023). DNMT1 regulates polarization of macrophage-induced intervertebral disc degeneration by modulating SIRT6 expression and promoting pyroptosis in vivo. Aging (Albany NY).

[193] Luo J., Jin G., Cui S., Wang H., Liu Q. (2024). Regulatory mechanism of FCGR2A in macrophage polarization and its effects on intervertebral disc degeneration. J Physiol.

[194] Wang Y., Gao Y., Cao Z., Dong M., Shao P., Fan H. (2026). NsPEFs-enriched ADSCs-EVs alleviate osteoarthritis via RSPO3-mediated dual pro-chondrogenic and pro-M2 macrophage properties. Bioact Mater.

[195] Chen Y.F., Lee C.W., Wu H.H., Lin W.T., Lee O.K. (2022). Immunometabolism of macrophages regulates skeletal muscle regeneration. Front Cell Dev Biol.

[196] Dort J., Fabre P., Molina T., Dumont N.A. (2019). Macrophages are key regulators of stem cells during skeletal muscle regeneration and diseases. Stem Cell Int.

[197] Feehan K.T., Bridgewater H.E., Stenkiewicz-Witeska J., De Maeyer R.P.H., Ferguson J., Mack M. (2024). Post-resolution macrophages shape long-term tissue immunity and integrity in a mouse model of pneumococcal pneumonia. Nat Commun.

[198] Liu Y., Ge M., Xiao X., Lu Y., Zhao W., Zheng K. (2025). Sarcosine decreases in sarcopenia and enhances muscle regeneration and adipose thermogenesis by activating anti-inflammatory macrophages. Nat Aging.

[199] Oishi Y., Koike H., Kumagami N., Nakagawa Y., Araki M., Taketomi Y. (2023). Macrophage SREBP1 regulates skeletal muscle regeneration. Front Immunol.

[200] Hoang D.H., Bouvière J., Galvis J., Moullé P., Mercier O., Migliavacca E. (2025). Immune aging impairs muscle regeneration via macrophage-derived anti-oxidant selenoprotein P. EMBO Rep.

[201] Zhao S., Wang J., Xue M., Wu B., Sheng L., Wen Y. (2026). Synovial inflammatory macrophage-derived extracellular vesicles exacerbate cartilage lesions with a FMRP-selectively sorted manner in osteoarthritis. Bone Res.

[202] Lu X., Zhang A., Zhang C., Guo D., Li Z.A., Xie D. (2026). Targeting the osteoporotic bone microenvironment: mechanistic insight and therapeutic biomaterials for accelerating bone regeneration. Bioact Mater.

[203] Wu W., Cheng Z., Shi P., Gao H., Chen X., Wu W. (2026). Microenvironment-educated MSC-EVs loaded injectable smart hydrogel for targeting senescent nucleus pulposus cells and inhibiting ferroptosis against intervertebral disc degeneration. Bioact Mater.

[204] Liu X., Carbajal E.E.P., Hwang Y.C., Mapkar S.A., Gilman B.W., Bliss S.A. (2026). Mesenchymal stromal cell-mediated intercellular communication: mapping the interactome for skeletal muscle homeostasis and regeneration. Adv Sci (Weinh).

[205] Xu H., Liu R., Zhou H., Kong B., Shen K., Zhao T. (2026). Engineered small extracellular vesicles as bioactive materials: integrating engineering strategies for cargo loading and targeted delivery systems. Bioact Mater.

[206] Xu Y., Ye Z., Wang Y., Ma Y., Chen X., Wang S. (2025). Alleviating osteoarthritis-induced damage through extracellular vesicles derived from inflammatory chondrocytes. Int Immunopharmacol.

[207] Li B., Shen E., Wu Z., Qi H., Wu C., Liu D. (2026). BMSC-Derived exosomes attenuate rat osteoarthritis by regulating macrophage polarization through PINK1/Parkin signaling pathway. Cartilage.

[208] Wang T., Zhao H., Zhang Y., Liu Y., Liu J., Chen G. (2024). A novel extracellular vesicles production system harnessing matrix homeostasis and macrophage reprogramming mitigates osteoarthritis. J Nanobiotechnol.

[209] Hu Y., Hou Z., Liu Z., Wang X., Zhong J., Li J. (2024). Oyster mantle-derived exosomes alleviate osteoporosis by regulating bone homeostasis. Biomaterials.

[210] Lei L.M., Li F.X., Lin X., Xu F., Shan S.K., Guo B. (2024). Cold exposure-induced plasma exosomes impair bone mass by inhibiting autophagy. J Nanobiotechnol.

[211] Huang C., Xiao Y., Qing L., Tang J., Wu P. (2025). Exosomal non-coding RNAs in the regulation of bone metabolism homeostasis: molecular mechanism and therapeutic potential. Heliyon.

[212] Lu L., Wang L., Wang H., Yang M. (2025). Regulatory role of miR-128-2-5p in serum exosomes on COL6A2 expression and postmenopausal osteoporosis. Hum Mol Genet.

[213] Kang M., Li Z., Chang I., Xu C., Chiang M., Kim L. (2024). Phosphatidylserine-incorporated exosome mimetics encapsulating CXCR3 antagonist alleviate osteoporosis. Adv Funct Mater.

[214] He Y., Chen Y. (2024). The potential of exosomes for osteoporosis treatment: a review. Drug Des Dev Ther.

[215] Hammoor B.T., Lai C.S., Xiong G.X., Elliott D.M., Snyder B., Vresilovic E. (2026). Intervertebral disc degeneration. Nat Rev Dis Primers.

[216] Li F., Shi Y., Liu J., Yang Y., Yan C., Sun X. (2026). An intelligent nanoliposome alleviates disc degeneration and discogenic pain by inhibiting neurovascular ingrowth via a "Soil-conditioning, seed-modulating, and weeds-suppressing" strategy. Bioact Mater.

[217] Xia P., Zheng J., Han Z., Chen Y., Xu L., Yu J. (2026). Lactate metabolism reprogramming through orthogonal tandem catalysis to reverse intervertebral disc degeneration. Nat Commun.

[218] Li L., Deng W., Chen S., Li Q., Xiao X., Long L. (2026). A context-aware artificial metabzyme in intervertebral disc degeneration metabolic convergence therapy. Nat Commun.

[219] He Y., Lin T., Liang R., Xiang Q., Tang T., Ge N. (2024). Interleukin 25 promotes muscle regeneration in sarcopenia by regulating macrophage-mediated Sonic Hedgehog signaling. Int Immunopharmacol.

[220] Sousa N.S., Brás M.F., Antunes I.B., Lindholm P., Neves J., Sousa-Victor P. (2023). Aging disrupts MANF-mediated immune modulation during skeletal muscle regeneration. Nat Aging.

[221] Zhu P., Kong F., Wu X., Dong Z., Du J., Mao Y. (2023). A minimally invasive Annulus Fibrosus Needle Puncture model of intervertebral disc degeneration in rats. World Neurosurg.

[222] Vadalà G., Russo F., Pattappa G., Schiuma D., Peroglio M., Benneker L.M. (2013). The transpedicular approach as an alternative route for intervertebral disc regeneration. Spine.

[223] Salzer E., Gorgin Karaji Z., van Doeselaar M., Tryfonidou M.A., Ito K. (2025). The role of loading-induced convection versus diffusion on the transport of small molecules into the intervertebral disc. Eur Spine J.

[224] Bianchini E., Ashley Sin YJ., Lee Y.J., Lin C., Anil U., Hamill C. (2024). The role of Hyaluronan/Receptor for Hyaluronan-Mediated motility interactions in the modulation of macrophage polarization and cartilage repair. Am J Pathol.

